# Characterization and comparative profiling of ovarian microRNAs during ovine anestrus and the breeding season

**DOI:** 10.1186/1471-2164-15-899

**Published:** 2014-10-15

**Authors:** Ran Di, Jianning He, Shuhui Song, Dongmei Tian, Qiuyue Liu, Xiaojun Liang, Qing Ma, Min Sun, Jiandong Wang, Wenming Zhao, Guiling Cao, Jinxin Wang, Zhimin Yang, Ying Ge, Mingxing Chu

**Affiliations:** Key Laboratory of Farm Animal Genetic Resources and Germplasm Innovation of Ministry of Agriculture, Institute of Animal Science, Chinese Academy of Agricultural Sciences, No. 2, Yuanmingyuan West Rd, Beijing, China; Core Genomic Facility, Beijing Institute of Genomics, Chinese Academy of Sciences, Beijing, China; Research Center of Grass and Livestock, Ningxia Academy of Agriculture and Forestry Sciences, Yinchuan, Ningxia China

**Keywords:** Sheep, Seasonal estrus, Anestrus, Ovary, miRNA, piRNA

## Abstract

**Background:**

Seasonal estrus is a critical limiting factor of animal fecundity, and it involves changes in both ovarian biology and hormone secretion in different seasons. Previous studies indicate that two classes of small RNAs (miRNAs and piRNAs) play important regulatory roles in ovarian biology. To understand the roles of small RNA-mediated post-transcriptional regulation in ovine seasonal estrus, the variation in expression patterns of ovarian small RNAs during anestrus and the breeding season were analyzed using Solexa sequencing technology. In addition, reproductive hormone levels were determined during ovine anestrus and the breeding season.

**Results:**

A total of 483 miRNAs (including 97 known, 369 conserved and 17 predicated novel miRNAs), which belong to 183 different miRNA families, were identified in ovaries of Tan sheep and Small Tail Han (STH) sheep. Compared with the three stages of the breeding season, 25 shared significantly differentially expressed (including 19 up- and six down-regulated) miRNAs were identified in ovine anestrus. KEGG Pathway analysis revealed that the target genes for some of the differentially expressed miRNAs were involved in reproductive hormone related pathways (e.g. steroid biosynthesis, androgen and estrogen metabolism and GnRH signaling pathway) as well as follicular/luteal development related pathways. Moreover, the expression of the differentially expressed miRNAs and most of their target genes were negatively correlated in the above pathways. Furthermore, the levels of estrogen, progesterone and LH in ovine anestrus were significantly lower than those in the breeding season. Combining the results of pathway enrichment analysis, expression of target genes and hormone measurement, we suggest that these differentially expressed miRNAs in anestrus might participate in attenuation of ovarian activity by regulating the above pathways. Besides miRNAs, a large and unexpectedly diverse set of piRNAs were also identified.

**Conclusions:**

The miRNA profiles of ovine ovaries in anestrus were presented for the first time. The identification and characterization of miRNAs that are differentially expressed between ovine anestrus and the breeding season will help understanding of the role of miRNAs in the regulation of seasonal estrus, and provides candidates for determining miRNAs which could be potentially used to regulate ovine seasonal estrus.

**Electronic supplementary material:**

The online version of this article (doi:10.1186/1471-2164-15-899) contains supplementary material, which is available to authorized users.

## Background

Many kinds of animal, such as sheep, horse, bird and fish, are seasonal breeders. Seasonal breeding is a critical limiting factor of animal fecundity. Most sheep in Northern China are estrous in the short-day season and anestrus in the long-day season [1]. Therefore, ewes cannot mate during anestrus and no lambs can be born for several months. Thus, for the mutton industry, seasonal estrus has been a bottleneck limiting efficient lamb meat supply throughout the year.

MicroRNAs (miRNAs) belong to a large family of endogenous non-coding RNAs and have emerged as important post-transcriptional regulators. Many miRNAs have been identified in hircine ovary [2, 3], mammary gland [4], testis [5], skin [6], hair [7], or ovine muscle [8]. Recently, genome-wide changes of miRNAs associated with follicular-luteal transition were identified in ovine ovary [9]. Previous studies in mice and pig indicated that miRNAs play critical roles in almost all ovarian biological processes [10–12]. For sheep, the obvious difference of ovarian biology exists between ovine anestrus and the breeding season. However the roles of miRNAs in the regulation of ovine seasonal estrus are unknown. Moreover, to date, only 153 (105 precursors) ovine miRNAs have been identified and listed in the miRBase database (Release 20). The characteristics of miRNA profiles in the ovine anestrus ovary are still unclear.

PIWI-interacting RNAs (piRNAs) are a recently discovered class of small non-coding RNA with a typical length between 23 nt and 32 nt [13–15]. They are typically clustered in the genome in a mono-directional or bi-directional manner. They are highly expressed in sperm cells and have also been identified in oocytes and ovaries of *Drosophila* and mammals [16–19]. The piRNA pathway is essential in early oogenesis and in transposon silencing in follicle cells [16, 20]. However, there has been no description of piRNAs in the ewe ovary until now.

To understand the roles of sRNA-mediated post-transcriptional regulation in ovine seasonal estrus, it is necessary to identify the differentially expressed small RNAs in ovine ovaries during anestrus and the breeding season. In Northern China, the Small Tail Han (STH) sheep is a famous local breed because of its year-round estrus and prolificacy. Tan sheep is another popular breed in Northern China because of its delicious meat and excellent lamb skin; however, it has a long anestrus stage usually from April to July. Therefore, the two breeds were considered ideal models for this study.

In this study, the expression profiles of small RNAs in ewe ovaries were compared between anestrus (A) and three distinct stages (luteal phase-L, proestrus-P and estrus-E) during the breeding season using Solexa sequencing technology. KEGG Pathway analyses were then implemented on target genes of the differentially expressed miRNAs. The correlation between the expressions of miRNA and their target genes in reproduction related pathways were analyzed. Since seasonal estrus involves changes in hormone secretion in different seasons, the reproductive hormone levels were also determined in Tan and STH sheep during anestrus and the breeding season. Collectively, these esults will help us to understand the role of miRNAs in the regulation of seasonal estrus.

## Results

### Dynamic changes of ovine hormone levels between anestrus and the breeding season

**Figure 1:**
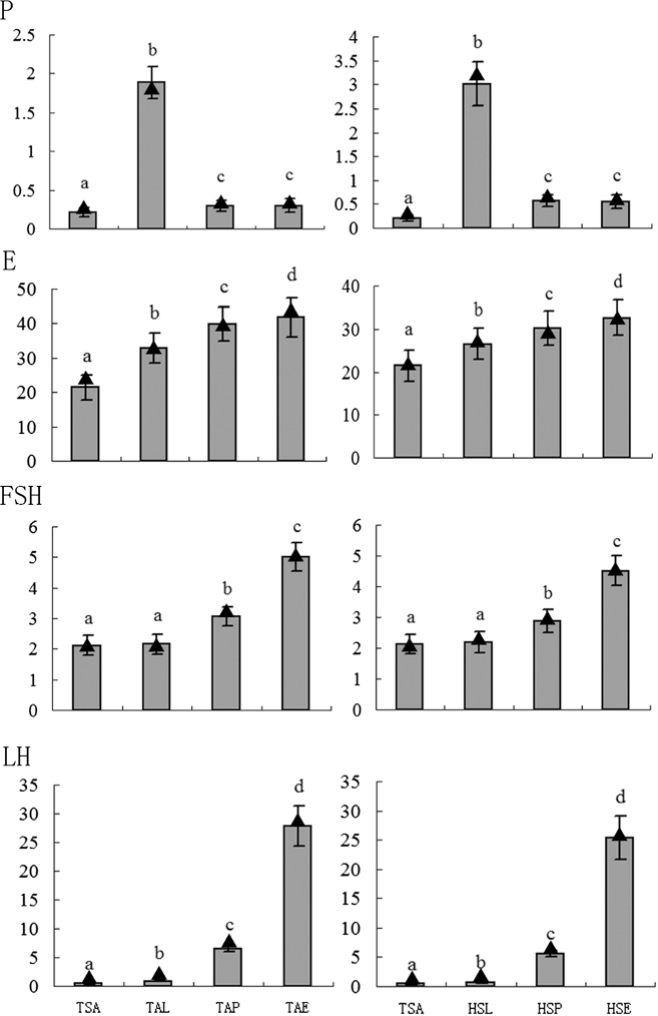
**Serum concentration of progesterone, estrogen, FSH and LH in different reproductive stages of Tan and Small Tail Han ewes.** Four hormones were measured in ng/mL, pg/mL, ng/mL and ng/mL, respectively. P: progesterone; E: estrogen. TSA: anestrous Tan ewes in spring; TAL: Tan ewes in luteal phase in autumn; TAP: proestrous Tan ewes in autumn; TAE: estrous Tan ewes in autumn; HSL: Small Tail Han ewes in luteal phase in spring; HSP: proestrous Small Tail Han ewes in spring; HSE: estrous Small Tail Han ewes in spring. Each Column represented mean value of each stage, and the par represented standard deviation. The different letters meant the significant difference among stages (*P* < 0.01). The triangles stood for the hormone concentration of samples used to perform RNA-seq.

Hormone fluctuation is a characteristic of reproductive status transition. The reproductive hormones levels in Tan and STH sheep were detected in anestrus and at different stages of the breeding season (Figure 1). Briefly, the levels of estrogen, progesterone and luteinizing hormone (LH) in anestrus were significantly lower compared to those in the breeding season (*P* < 0.01). Specifically, the secretion patterns of the four hormones were different. A high concentration of estrogen can promote estrus while a low level inhibits estrus. Our results showed that its secretion gradually increased from anestrus to the luteal phase, proestrus and estrus. Progesterone is a hormone secreted mainly by the corpus luteum, and it showed the lowest level in anestrus and the highest level in the luteal phase. Follicle-stimulating hormone (FSH) and LH are two important endocrine regulators of follicular growth, development and ovulation. Our results demonstrated that their concentrations in anestrus were significantly lower (*P* < 0.01) than those in proestrus and estrus. The hormone results also confirmed that our samples used to perform RNA-seq were collected from the right stages.

### Overview of Solexa-sequencing of ovine ovary small RNAs

**Figure 2:**
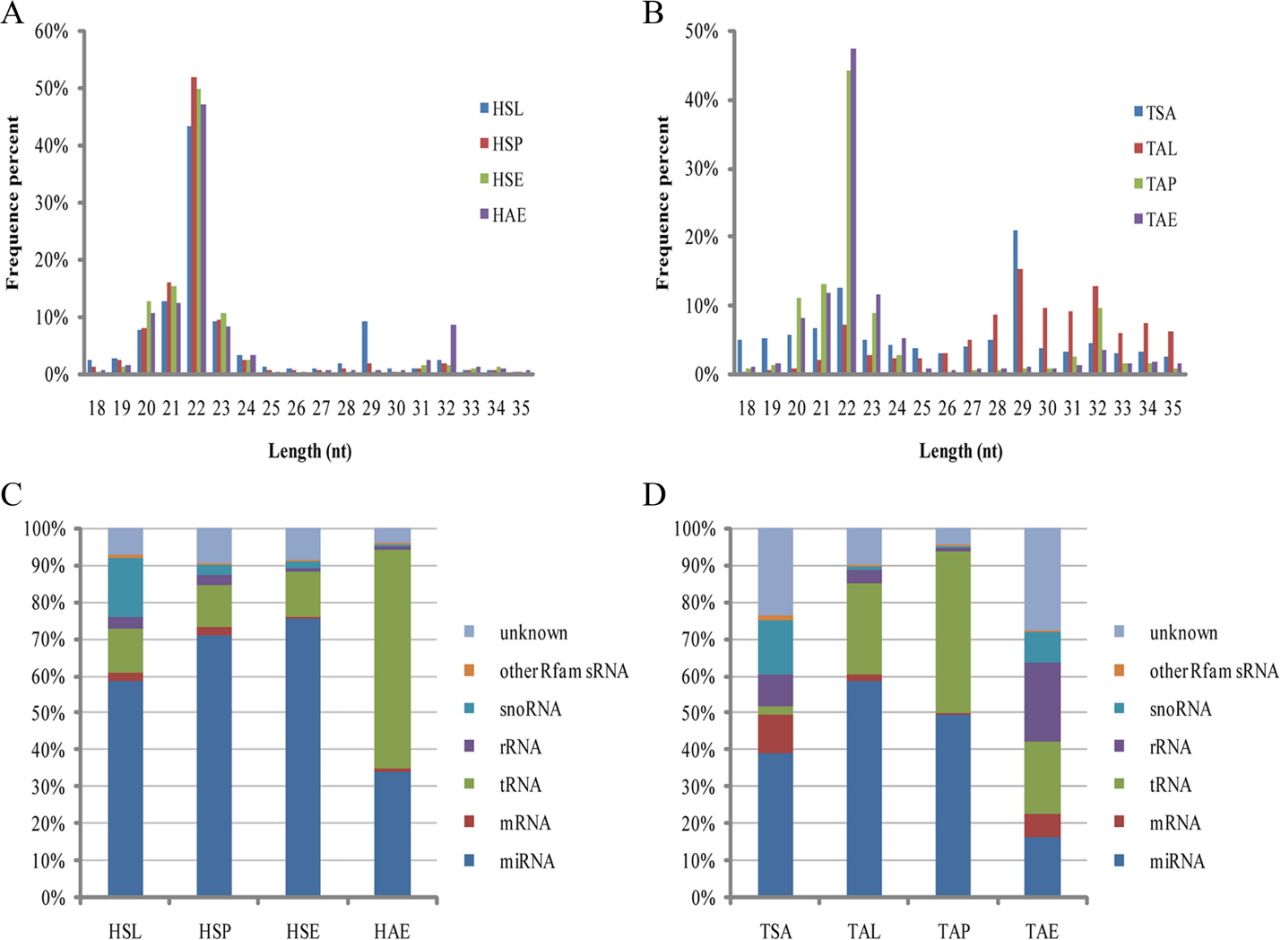
**Sequence distribution of mapped reads.** Frequency distribution of sequence length of STH sheep **(A)** and Tan sheep **(B)** based on the abundance of mapped reads. The composition of the RNA classes in each library was shown for STH sheep **(C)** and Tan sheep **(D)**.

To understand the expression pattern of ovarian small RNAs in anestrus and the three stages during the breeding season, eight sRNA libraries representing the different stages were constructed from eight adult Tan and STH sheep ovary samples [including: anestrous Tan ewes in spring (TSA), Tan ewes in luteal phase in autumn (TAL), proestrous Tan ewes in autumn (TAP), estrous Tan ewes in autumn (TAE); STH ewes in luteal phase in spring (HSL), proestrous STH ewes in spring (HSP), estrous STH ewes in spring (HSE), estrous STH ewes in autumn(HAE)]. The small RNA libraries were subsequently sequenced and a total of 156,694,170 raw reads were obtained. After removing adapters and discarding sequences shorter than 17 nt or longer than 35 nt, 128,389,756 clean reads remained (Additional file 1) for further analysis. For all samples, 65-89% of the clean distinct tags (unique sRNAs) for each library were retained by clustering the same reads and aligning to the sheep genome (NCBI, Oarv3.1). For example, in 9,062,723 clean reads of the TSA sample, 7,049,153 reads representing 77.78% of total clean reads were aligned to the sheep genome sequence (Additional file 1). In STH sheep, the mapped reads demonstrated a single-peaked length distribution with a peak at 22 nt (Figure 2A) and in Tan sheep, a length distribution with three peaks at 22 nt, 29 nt and 32 nt (Figure 2B). The 22 nt length class was consistent with the common size of miRNAs, whereas the 29 nt and 32 nt classes might represent piRNAs. To further assess the efficiency of Solexa sequencing for miRNA detection, all of the mapped clean reads were annotated and classified by aligning against non-coding RNAs (ncRNAs) in the Rfam database and miRbase 20.0. For all samples except HAE and TAE, most sRNA sequences were clustered into miRNA sequences (Figure 2C, D). In addition to the highly enriched miRNA sequences, there were also lowly expressed but much more diverse sequences of other ncRNAs, such as rRNAs, tRNAs, mRNAs, snoRNAs and other sRNAs in the Rfam database (Figure 2C, D).

### Expression analysis of miRNAs and miRNA families in ovine ovary

**Figure 3:**
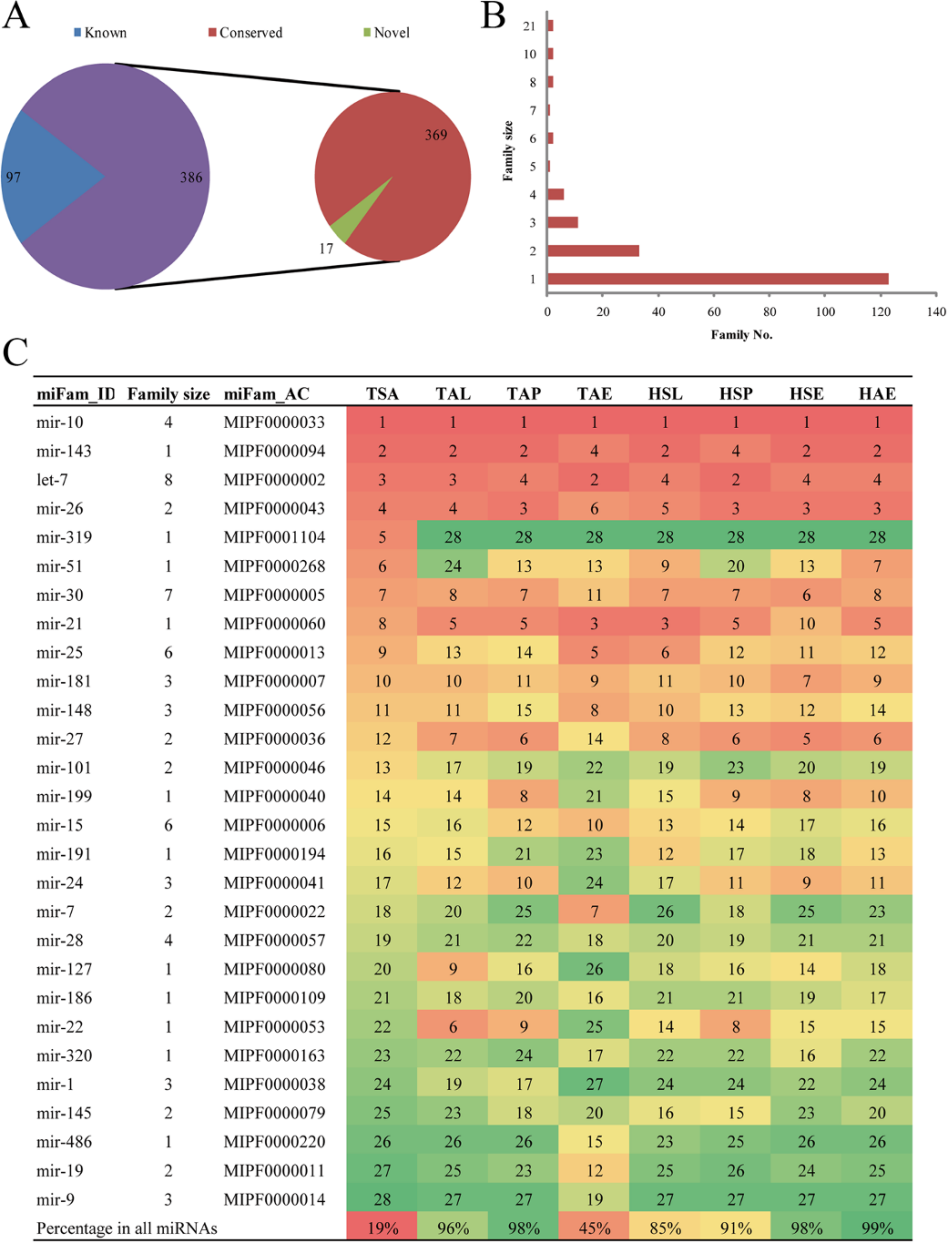
**Expressed miRNAs and miRNA families. (A)** The number of expressed miRNAs, including known, conserved and predicated novel miRNAs; **(B)** Distribution of miRNA family size; **(C)** The top ranked 20 expressed miRNA families in each sample.

A total of 483 miRNAs were identified in ovine ovary, including 97 known miRNAs, 369 conserved miRNAs and 17 predicated novel miRNAs (Figure 3A). All these miRNAs were classified into 183 miRNA families based on an established miRNA family system (the miRBase family organization). The size of miRNA families varied from 1 to 21 (Figure 3B). Specifically, 123 (67%) miRNA families had a single member, 33 (18%) had two members, and the mir-154 and mir-2284 families had the most members (N = 21). It is noteworthy that different members in the same miRNA family might display drastically different expression levels. For example, the expression abundance of let-7 family members varied from 0 to 344,318 reads. The 20 most highly expressed miRNA families were summarized for each sample (Figure 3C). The mir-10 (MIPF0000033), mir-143 (MIPF0000094), let-7 (MIPF0000002) and mir-26 (MIPF0000043) families were found to be preferentially expressed in ovine ovary. In addition to the above four families, the mir-319 (MIPF0001104) family was highly expressed in the TSA sample. The sum of miRNA abundance of the top 20 miRNA families represented more than 85% of total sRNAs in most samples, while this percent only reached 19% in the TSA sample.

### Expression profile of ovarian miRNAs in ovine anestrus

**Figure 4:**
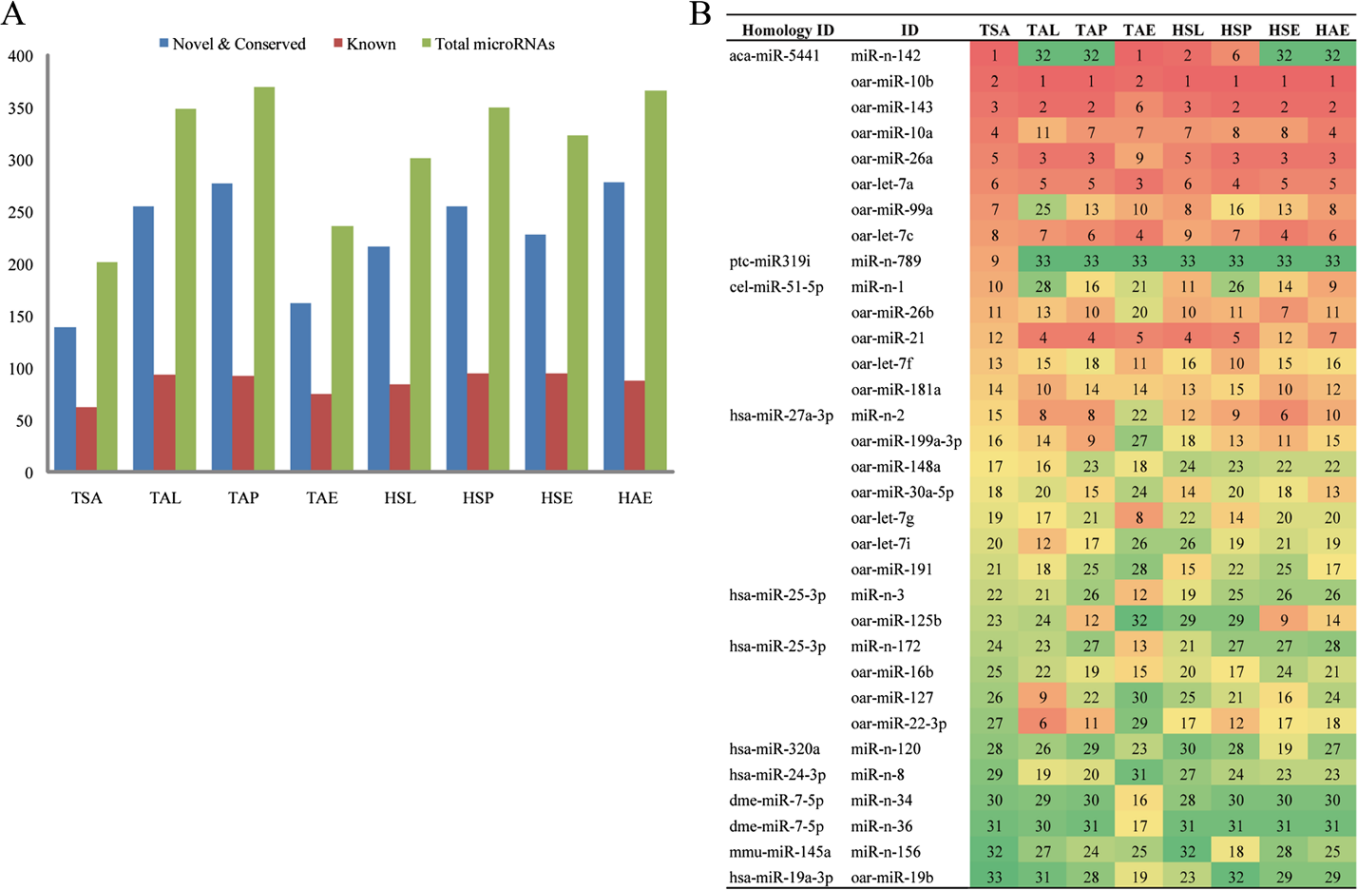
**Comparison of the miRNA expression profiles between anestrus and the other three stages. (A)** Expressed miRNAs for each sample; **(B)** The top expressed miRNAs in each sample.

In this study, the whole expression profile of ovarian miRNAs in ovine anestrus was determined for the first time. In total, 202 expressed miRNAs, including 63 known, 136 conserved and three novel predicated miRNAs were identified (Figure 4A). Compared to the other three stages, the number of expressed miRNAs is the smallest and the total abundance of the top 20 miRNA families is approximately 19% of all expressed sRNAs in anestrus stage sample, which suggests that novel miRNAs were highly expressed in anestrus. Therefore, miRNAs in each sample were also ranked based on their level of expression (Figure 4B). In the 33 most highly expressed miRNAs, miR-n-142 (homology ID: aca-miR-5441), a newly identified conserved miRNA, was the most abundant miRNA in anestrus which accounted for 82% of the total expressed miRNAs. KEGG pathway analysis showed that the target genes of miR-n-142 were predominantly enriched in oxidative phosphorylation, glycerolipid metabolism and phosphatidylinositol signaling pathways. miR-n-789 (Homology ID: ptc-miR319i) is another remarkably highly expressed miRNA in anestrus, and its target genes were enriched in circadian rhythm, notch signaling and the GnRH signaling pathway. The other highly expressed miRNAs, such as oar-let-7a, oar-miR-21 and oar-miR-125b have been reported to be highly expressed in ovine ovarian follicles [2]. The newly identified highly expressed miRNAs in anestrus will facilitate understanding of their new roles in seasonal estrus.

### Comparative analysis of the differentially expressed miRNAs between anestrus and the other three stages during the breeding season

**Figure 5:**
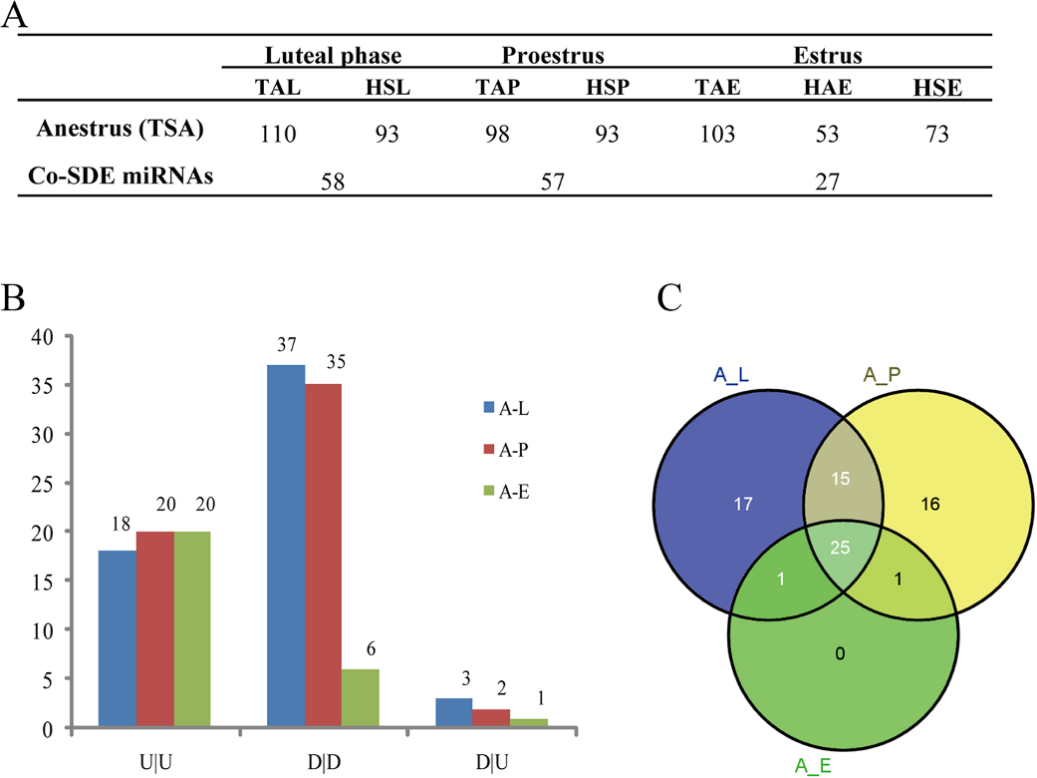
**Differentially expressed miRNAs between anestrus and the other stages during estrus cycle. (A)** The number of differentially expressed miRNAs. **(B)** The number of down-regulated miRNAs (indicates in D) and up-regulated (indicates in U) genes in anestrus when compared to the other three stages during estrus cycle. A-L: anestrus *vs.* luteal phase; A-P: anestrus *vs.* proestrus; A-E: anestrus *vs.* estrus. “U/U” and “D/D”: the expression patterns (up-regulation or down-regulation) of miRNAs between anestrus of Tan and the other stages of Tan were basically consistent with those between anestrus of Tan and the other stages of STH sheep; “D/U” means differentially expressed miRNAs are inconsistent between above two comparisons. **(C)** Venn diagram showed the 25 overlapped differentially expressed miRNAs between anestrus and the other three stages.

**Figure 6:**
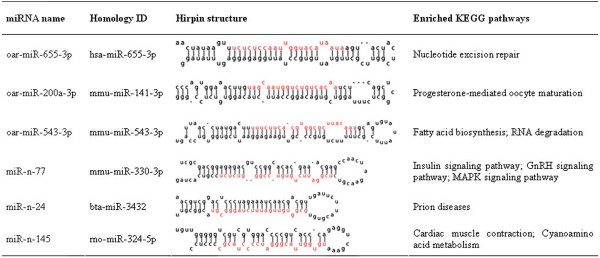
**Hirpin structure and enriched KEGG pathways of significantly down-regulated miRNAs in anestrus.**

**Figure 7:**
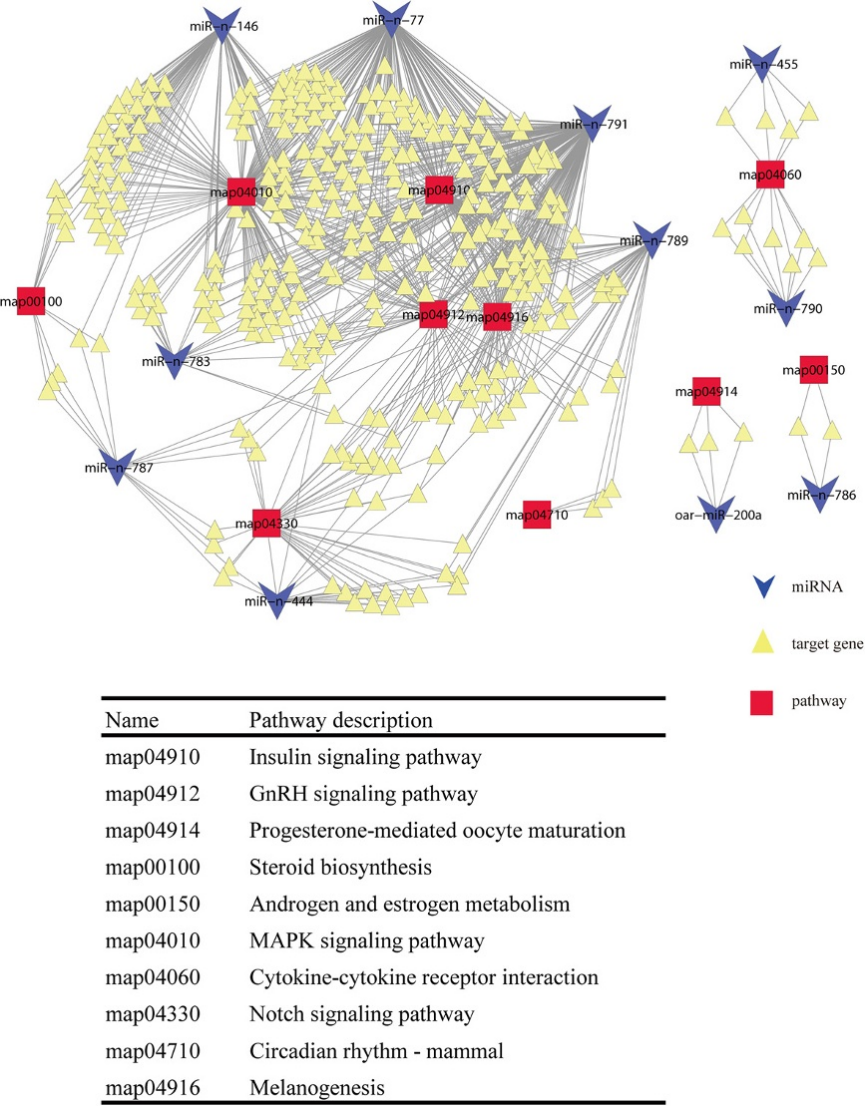
**Network consisting of differentially expressed miRNAs, their target genes and enriched KEGG pathways.**

The ovarian miRNA expression profile in anestrous Tan sheep was compared with those in the other three stages in both Tan and STH sheep (Figure 5A). In total, 58, 57 and 27 significantly differentially expressed miRNAs were identified between anestrus *vs.* luteal phase, anestrus *vs.* proestrus and anestrus *vs.* estrus, respectively. For these significantly differentially expressed miRNAs, their expression patterns (up- or down-regulation) between anestrus of Tan and the other stages of Tan were basically consistent with those between anestrus of Tan and the other stages of STH sheep (Figure 5B). With the aim of intensively identifying miRNAs associated with anestrus, we finally focused on 25 shared miRNAs that were significantly differentially expressed between anestrus and other three stages (Figure 5C). Of the 25 differentially expressed miRNAs, 19 were significantly up-regulated and 6 were significantly down-regulated in anestrus. The precursor sequences and chromosome locations of the 14 most highly up-regulated miRNAs were predicated and are shown in Table 1. Hairpin structures of six significantly down-regulated miRNAs are shown in Figure 6. To understand the function of these differentially expressed miRNAs, KEGG pathway analysis was performed. Some target genes of these miRNAs were enriched in reproduction related pathways (Table 1 and Figure 6), such as the reproductive hormone related pathways (e.g. androgen and estrogen metabolism, steroid biosynthesis and GnRH signaling pathway) as well as follicular (or luteal) development related pathways (e.g. Notch and MAPK signaling pathway and progesterone-mediated oocyte maturation). Simultaneously, the expression levels of predicted target genes of the differentially expressed miRNAs were extracted and analyzed from RNA-sequencing results (un-published). By correlation analysis, we found that the expression levels of these differentially expressed miRNAs were negatively correlated with most of their target genes in the above pathways (Additional file 2). Finally, a network that is composed of the differentially expressed miRNAs, their target genes and enriched KEGG pathways is shown in Figure 7. Some miRNAs shared the same target genes or reproduction related pathways.

**Table 1:** **Precursor sequences, genome locations and enriched KEGG pathways of significantly up-regulated miRNAs in anestrus**

miRNA	Chromosome	Start	End	Strand	miRNA precursor sequence	Enriched KEGG pathways
miR-n-783	chr26	38599891	38599941	+	ccaccgggcuccucugaccacgggacuucccagagaagacugcagugggu	MAPK signaling pathway; GnRH signaling pathway; Oxidative phosphorylation
miR-n-786	chr21	8878698	8878742	+	uugguguaugugcuuggcugagaagccaauggggcgaagcuacc	Androgen and estrogen metabolism; Fatty acid metabolism
miR-n-784	chr7	84685572	84685648	-	ccacggacuguacaguccuuggggcugcaaagagucggacaggacugagcucuuucaggaaugacaguccccaguc	Basal transcription factors; Fatty acid biosynthesis
miR-n-787	chr16	496440	496499	+	cugggcuccucugucugaggugacaaugugucaaagucccagcaagagagaguccacag	Notch signaling pathway; Steroid biosynthesis
miR-n-444	chr16	39273458	39273523	-	caguucccucuuccuugccaguggagaugacagugcucuccaagaagcuggaggggaggacuucc	Notch signaling pathway; Apoptosis
miR-n-782	chr2	111819172	111819244	+	cguggaucaaagcaucuauggauuucccucgugucuuucccacgaggcuuuccaacgaggcuuucccacagg	Metabolism of xenobiotics by cytochrome P450
miR-n-793	chr4	118592936	118593008	-	cguggaucaaagcaucuaugguuuucccucgugucuuucccacgaggcuuucccacgaggcuuucccacagg	Metabolism of xenobiotics by cytochrome P450
miR-n-792	chr16	38836730	38836775	-	uccuuuguaaacaucuggaaagccaagcuguaaacucugaggauc	-
miR-n-790	chr24	5171923	5171991	-	ccauggcuugguucaauacucuuaaguuacuccucaacccauggaagacuaggggacuguuauguguc	Cytokine-cytokine receptor interaction; MAPK signaling pathway
miR-n-785	chr3	142054999	142055066	-	gcauuaugguuacauuguggcugcugcuacagcucuuguuuccgccaggauggagacuguguuaugg	Basal cell carcinoma; PPAR signaling pathway
miR-n-788	chr26	232395	232460	+	cagaguugaggcugggagcuccagggcuaagguccugggaccaggcucugggucucugacuuggg	Long-term depression
miR-n-791	chr5	4904837	4904905	+	ucaggggcaauggccagguggagcugguguggucaguucuggggcuccugggccgugugccccuggac	Insulin signaling pathway; GnRH signaling pathway; MAPK signaling pathway; Notch signaling pathway
miR-n-795	chr19	52910579	52910655	+	ccagcauugaggggccaguggaauucuggugcaggcucucucccuggcuugccaguggucacuuucuugcuguguc	MAPK signaling pathway
miR-n-794	chr2	175146052	175146125	+	caaugagggacuggaccaguuggauucugggguaagaaugcucuggguggaaguacuagcuagaacccaggcu	-

To verify the Solexa sequencing data, five miRNAs (miR-n-13, miR-n-162, miR-n-180, oar-miR-374b and miR-n-17) were randomly selected and qRT-PCR for all reproductive stages was conducted. There was a strong positive correlation (*R*^*2*^ = 0.975) between RNA-seq and qRT-PCR data indicating consistency of results.

### Predication of piRNAs from ovine ovary Solexa sequencing data

**Figure 8:**
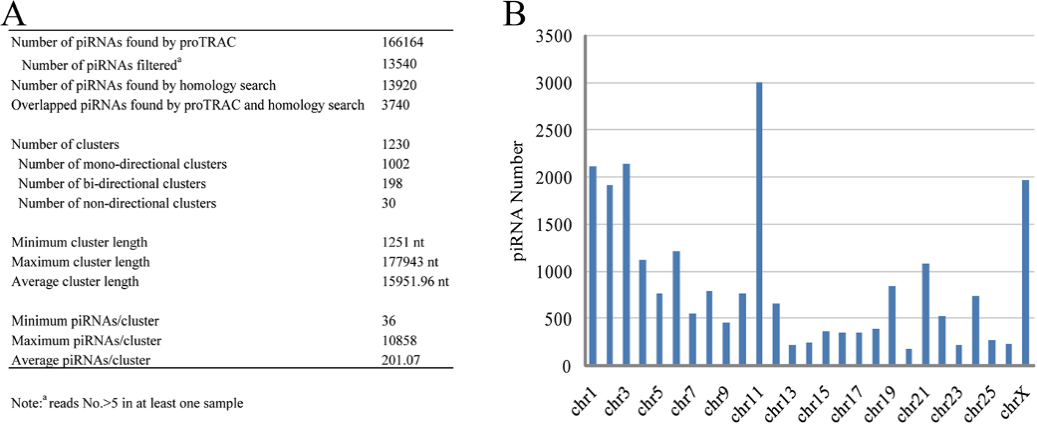
**Predicated piRNAs and piRNA clusters. (A)** Statistics of predicated piRNAs **(B)** chromosome distribution of predicated piRNAs with reads numbers >5 in at least one sample.

piRNA pathways are particularly essential for genome integrity, retrotransposon repression and germ cell development. piRNAs have been identified in CHO cells and oocytes of mouse [21, 22]. In our ovine ovary sequenced libraries, a distinct subset of small RNAs ranging from 24 to 33 nt was detected and they were derived from ewe genomic regions that do not encode miRNAs. These small RNAs were characterized using two methods. By *de novo* prediction, a total of 166,164 (23,288 piRNAs with read numbers > 5) candidate piRNAs were predicated (Figure 8A). Homology searches against to known piRNAs in piRNABank identified 13,920 homologous piRNAs (Figure 8A). Of all of the predicated piRNAs, 3,740 sequences were also identified by homology search. These piRNAs displayed a highly uneven distribution among chromosomes and were enriched on chromosomes 11, 1, 2, 3 and X (Figure 8B). The predicated piRNAs came from 1,230 piRNA cluster loci. Each cluster locus contained 36–10,858 piRNAs (an average of 201 piRNAs) and spanned from 1,251 to 177,943 bp (an average of 15,951 bp). According to the definition of piRNA cluster types described by proTRAC, 1,002 clusters were mono-directional while 198 clusters were bi-directional and distributed on two strands, and only 30 clusters had no directional orientation.

## Discussion

Tan and STH sheep are two well known breeds in Northern China, and they are seasonal and year-round breeders, respectively. To understand the roles of sRNA-mediated post-transcriptional regulation in ovine seasonal estrus, we compared the small RNA profiles of ovarian tissues between anestrus of Tan sheep and three stages during the breeding season of both Tan and STH sheep using Solexa sequencing technology.

### miRNAs with high expression levels in anestrus and other stages during the breeding season

The information in the ovine miRNA database has been enriched by this study and this is the first report of miRNA expression profiles of anestrous ovine ovary tissues. Some of the abundant miRNAs in the anestrous ovary of Tan sheep, including miR-143, miR-26a, let-7 and miR-21, were also reported to be highly abundant in ovaries of human, cow [23–25], pig [26], adult and neonatal mouse [27, 28] and sheep [2, 3]. Moreover, according to our results, these miRNAs were also highly expressed in ovine ovaries of different stages during the breeding season. This conserved high level of expression across mammalian species and reproductive stages suggests their important roles in mammalian ovarian function. In previous studies, miR-21 was identified to be able to promote follicular cell survival during ovulation [29], miR-143 was critical for the formation of mouse primordial follicles [30] and let-7b was shown to be necessary for normal development of the corpus luteum [10]. Additionally, some KEGG pathways, in which the target genes of these miRNA were enriched, are important for ovarian function. For instance, miR-143 was significantly enriched in the GnRH signaling and progesterone-mediated oocyte maturation pathways, which are crucial for ovarian activity by influencing endocrine function and follicular development. In addition, the apoptosis, Wnt signaling, and C21-Steroid hormone metabolism pathways, which contain target genes of the let-7 family, are important for granulosa cell proliferation, follicular growth and development, and luteinic formation and regression. More importantly, miR-n-142 (homology ID: aca-miR-5441), a novel predicated conserved miRNA, was the most highly expressed miRNA in anestrus (accounting for 82% of total miRNA expression). Its target genes were mainly involved in oxidative phosphorylation, glycerolipid metabolism, and phosphatidylinositol signaling pathways. Further research is needed to reveal its potential role in reproduction.

### Differentially expressed miRNAs between anestrus and the other three stages in the breeding season

Besides the miRNAs with high levels of expression in anestrus, 25 miRNAs that were differentially expressed between anestrus and the other three stages were also identified, and most of them were novel. Of these, only the function of miR-n-145 had been studied and it is mainly involved in the regulation of proliferation of granulosa cells. Yan et al. reported that miR-n-145 mediated attenuation of activin-induced proliferation of mouse granulosa cells by targeting both activin receptor 1B and cyclin D2 [31]. When cultured bovine granulosa cells were treated with forskolin (an adenylate cyclase agonist) to promote luteinization, a decrease in the levels of miR-n-145 was observed during the follicular-luteal transition [2]. These results implied that miR-n-145 might mediate inhibition of granulosa cell proliferation during the follicular stage. During the ovine breeding season, the ovary is in the estrus cycle, including the follicular stage and luteal phase, so it is likely that the high level of miR-n-145 expression was involved in attenuating the proliferation of ovine granulosa cells. However, during anestrus the physiological activity of the ovary was relatively reduced and the follicular stage does not exist. Thus the proliferation of granulosa cells is significantly attenuated. Therefore, in our study, the low level of miR-n-145 expression observed in anestrus is reasonable.

### Reproduction-related pathways in which differentially expressed miRNAs were enriched

The KEGG pathway analysis indicated that some target genes of the differentially expressed miRNAs were enriched in reproduction-related pathways (Figure 7). First, some pathways were crucial for the secretion of gonadal hormones, and there were some successive relationships among the pathways. Specifically, both miR-n-787 and miR-n-786 were significantly enriched in estrogen–related pathways (Androgen and estrogen metabolism pathway and Steroid biosynthesis pathway), and the target genes of miR-n-791 were significantly enriched in the Insulin signaling pathway. As Additional file 3 shows, the Insulin signaling pathway may act on the estrogen–related pathway and ultimately they can regulate the biosynthesis of gonadal hormone (e.g. estrogen and progesterone) together. According to the RNA-sequencing results, miR-n-787 expression was negatively correlated with that of 80% of its target genes in the Steroid biosynthesis pathway; while the expression of miR-n-786 was negatively correlated with that of target genes (NSUN4 and UGT2C1) in the Androgen and estrogen metabolism pathway. Previous studies have indicated that the ewes in anestrus had relatively lower estrogen and progesterone levels compared with in the breeding season. In anestrus, progesterone secreted by the corpus luteum is suppressed because fewer ovulations led to a reduction of the total luteal volume; although it may also result from diminished gonadotropic support (e.g. LH). Our results for hormone determination also indicated that the level of estrogen and progesterone in anestrus of Tan sheep were significantly lower than those in the breeding season of Tan and STH sheep. These results suggest that miR-n-786 and miR-n-787 might play roles in the estrogen–related pathways by negatively regulating their target genes.

Second, the enriched KEGG pathways of differentially expressed miRNAs are important for follicular and luteal development. Targets of both miR-n-787 and miR-n-789 were enriched in the Notch signaling pathway, which is necessary for follicular development [32, 33] and vessel formation in the follicle and corpus luteum [34]. Correlation analysis indicated that the expression of miR-n-787 and miR-n-789 was negatively correlated with most of their target genes involved in the Notch signaling pathway. Trombly et al. [33] reported that the suppression of Notch signaling with gamma-secretase inhibitors in the neonatal mouse ovary could decrease primordial follicle formation. Complete blockage of the Notch signaling pathway with compound E also impairs folliculogenesis and induces disruption of gonadotropin stimulated angiogenesis [32]. Vorontchikhina et al. suggested that Notch signaling plays an important role in angiogenic growth during mouse folliculogenesis and in the corpus luteum [34]. In the breeding season, the vessels are rich in the antral follicle and corpus luteum; however, in anestrus, little vessel formation occurs because of the absence of ovulation and the corpus luteum. Collectively, these results indicate that miR-n-787 and miR-n-789 might play regulatory roles during vessel formation in the follicle and corpus luteum via the Notch signaling pathway. Another important pathway for follicular development is the MAPK signaling pathway, and target genes of miR-n-783, miR-n-791 and miR-n-77 were enriched in this pathway. The activation of the MAPK pathway could promote leptin-stimulated oocyte maturation, and conversely the maturation could be blocked when leptin-induced MAPK phosphorylation was suppressed by a specific MAPK activation inhibitor [35]. In addition, the Progesterone-mediated oocyte maturation pathway, in which the target genes of oar-miR-200a-3p were enriched, was related to maturation of the oocyte [36].

Third, it was interesting and unexpected that the target genes of miRNAs including miR-n-783, miR-n-791 and miR-n-77 were simultaneously enriched in the GnRH signaling pathway. In addition, some pathways relating to the GnRH signaling pathway were also involved; for example, the target genes of miR-n-790 were enriched in the Cytokine-cytokine receptor interaction pathway, and target genes of miR-n-783, miR-n-791 and miR-n-77 were synchronously enriched in the MAPK signaling pathway. It is noteworthy that these pathways could connect mutually in a network (Additional file 4) and finally could regulate the secretion of gonadotropins (FSH and LH), which are known as the crucial endocrine regulators of follicular growth, development and ovulation. Recent studies in plants, animals and humans show that miRNAs might regulate gene expression in other cells or tissues by migration to target cells or tissues [37–41]. Hence it is likely that, similar to how hormones secreted from the ovary can exert a feedback regulation on gonadotropins (FSH and LH) in the hypophysis, these miRNAs might be involved in similar regulation of gonadotropins by targeting genes in the above pathways. It has been reported that a low level of LH is necessary for anestrus [42, 43]. The hormone levels in Tan and STH ewes also showed that the concentration of LH in Tan anestrus was significantly lower compared to that in the breeding season (Figure 1). Collectively, these differentially expressed miRNAs in ovine anestrus might mediate post-transcriptional regulation on secretion of LH by the GnRH signaling pathway.

Finally, these findings provide candidates for further experimentation to identify miRNAs which could be potentially used to regulate ovine seasonal estrus.

### Predicted piRNAs in the ovine ovary

piRNAs are a new class of small RNAs that bind to the PIWI argonaute protein family. Large scale sequencing for piRNAs from rat, mouse, fly and human has yielded very high numbers of piRNA sequences, which can be acquired from piRNABank [44]. The piRNA-PIWI complexes have been reported to be involved in post-transcriptional gene silencing of retrotransposons and other genetic elements in germ line cells, particularly during spermatogenesis [45]. They were first discovered in spermatogenic cells [46] and testicular cells in rat [15]. piRNA also exist in mammalian ovaries [21, 47] and the piRNA pathway is essential for early oogenesis [48] and transposon silencing in follicle cells [16, 20]. Recently, it was found that piRNAs have widespread expression in all macaque tissues [49]. Usually, the length of piRNAs is concentrated at 26–31 nt. In our study, the length distribution of small RNAs showed double-peaks at 21–24 and 24–32 nt, and a considerable percentage (33%-69%) of reads were between 24 and 32 nt in length. Because of the lack of identified or previously reported piRNAs in ewe, in the present study, 166,164 candidate piRNAs of 24–33 nt were first identified using *de novo* prediction and homology search methods, and they were classified into 1,251 clusters. The number of piRNAs was comparable to the number of piRNAs previously found in other organisms listed in the piRNABank database. In our study the piRNAs in the ovine ovary displayed a highly uneven distribution among chromosomes and were enriched on chromosome 11 and X. This characteristic has also been reported in porcine testes [50]. Widespread piRNA expression across all reproductive stages hinted at their important functions in ovarian activity.

## Conclusions

For the first time, the miRNA profiles of the ovine ovary in anestrus are presented. In total, 483 miRNAs were identified in the ovine ovary. The expression patterns of the miRNAs varied between different reproductive stages. Compared with the three stages of the breeding season, 25 shared significantly differentially expressed (including 19 up- and six down-regulated) miRNAs were identified in ovine anestrus. Combining the results of pathway enrichment analysis, expression of target genes and hormone measurement, these differentially expressed miRNAs in anestrus are indicated to participate in attenuation of ovarian activity by regulating the pathways relating to hormone secretion and follicular development. In addition, a large and unexpectedly diverse set of piRNAs sequences were identified in the ewe ovary. Our findings will help understanding of the role of miRNAs in the regulation of seasonal estrus, and provides candidates for identifying miRNAs which could potentially be used to regulate ovine seasonal estrus.

## Methods

### Experimental animal tissue collection and hormone determination

All procedures involving animals were approved by the Animal Care and Use Committee of Chinese Academy of Agricultural Sciences, Beijing, China and the Animal Care and Use Committee of Ningxia Academy of Agriculture and Forestry Sciences, Yinchuan, Ningxia, China.

Tan sheep and STH sheep were selected from respective breed conservation farms and housed in the same farm in Ningxia Autonomous Region, China. The three years-old, clinically normal and non-pregnant Tan and STH ewes were examined daily for estrous activity with a teaser ram during four seasons. The date of estrous cycle and duration of estrus were recorded and their blood was collected daily for measurement of serum hormone concentrations. Estradiol, FSH and LH were measured by radioimmunoassay methods which have been described in detail [51–53]. Progesterone was measured using a radioimmunoassay method described previously [54] as validated for sheep [55]. Estrus was judged according to the obvious estrous signs in response to the teaser ram. Anestrus was a period without obvious estrous signs during more than 36 days (the time of two estrous cycles). Ewes in luteal phase and proestrus were determined according to records of three consecutive estrous cycles and the method of characterizing particular stages of the estrous cycle by plasma progesterone and LH concentrations [56]. Finally, eight ewes standing for different reproductive stages were selected randomly and killed for ovary collection. All ovary samples were immediately snap-frozen in liquid nitrogen for total RNA extraction.

Reproductive hormone (estrogen, progesterone, FSH and LH) levels in different reproductive stages were also analyzed with the above described radioimmunoassay methods in five Tan and five STH 3 year-old ewes. In anestrus, the blood was collected three times a day for18 days. During an integral estrus cycle (about 18 days) in the breeding season, for both Tan and STH ewes, blood was collected three times a day in luteal stage and proestrus, and once every two hours in estrus. For each hormone, the mean concentration in each stage was calculated and the difference among stages was analyzed using the F-test program of SAS 8.0.

### RNA extraction, mRNA and small RNA sequencing

Total RNA was extracted from ovary tissue at different stages using TRIzol (Invitrogen Carlsbad, CA, USA) according to the manufacturer’s instruction.

Low-molecular weight RNAs (<40 nucleotides long) were isolated from total RNA using a FlashPAGE fractionator (Ambion, Life Technologies, Paisley, UK). miRNA libraries were constructed using the Illumina TruSeq Small RNA Sample Preparation kit, in which miRNAs were ligated to two adapters (5′ Adapter: 5′-GUUCAGAGUUCUACAGUCCGACGAUC-3′; 3′ Adapter: 5′-TGGAATTCTCGGGTGCCAAGG-3′) and amplified by RT-PCR, the amplification products (140–160 bases, including the small RNA and adapter sequences) were further purified on a 15% polyacrylamide TBE gel and sequenced with an Illumina Hiseq 2000 system. The mRNA libraries for different reproductive stages were generated using Illumina Truseq RNA Sample Preparation Kits. The required fragments were enriched by PCR amplification and purified using a Qiagen MiniElute PCR Purification Kit. The library products were sequenced with an Illumina HiSeq 2000 system. The library construction and sequencing of miRNAs and mRNAs was performed at Beijing Institute of Genomics, Chinese Academy of Sciences (Beijing, China).

### Bioinformatic analysis of sequenced small RNAs

All sequenced raw fastq reads from each small RNA library were used for miRNA analysis. First, the 3′ primer adaptor sequences were removed by the fastx clipper tool (http://hannonlab.cshl.edu/fastx_toolkit). Then, all reads were clustered into one tag and those tags shorter than 18 nucleotides were filtered using an in-house built pipeline [57]. Finally, the mirDeep2 program [58] was used for miRNA predication. With mirDeep2, clean tags were first aligned to the sheep genome (NCBI, Oarv3.1) using mappper.pl script without any nucleotide changes (genome hits < 5); a small RNA tag was defined as a miRNA (score > 0) through evaluation of both expression level and biogenesis criteria using miRDeep2.pl scripts. All the mapped tags were annotated by comparison with the ncRNAs (rRNAs, tRNAs, snRNAs, and snoRNA) deposited in the NCBI GenBank database and the Rfam10.1 database using the BLAST program.

All predicated miRNAs were classified into novel (which were not present in MiRbase), known (known ovine miRNAs in MiRbase) and conserved (known miRNAs of other species in MiRbase but have not been found in sheep) miRNAs based on miRbase-v20, and were also simultaneously classified into different miRNAs families based on an established miRNA family system in miRBase-v20 using in-house built Perl scripts. The miRNA expression in each library was normalized as FPM values (reads per million), which was calculated as follows: number of sequenced reads/total mapped reads × 1,000,000. The differentially expressed microRNAs among different stages were obtained by the edgeR software package [59] which used an empirical Bayesian method to test differential expression in deep sequencing datasets, and during this process the sequencing depth was normalized using edgeR software. Those miRNAs with *p*-values <0.001 were defined as differentially expressed.

### Bioinformatic analysis for sequenced mRNAs

For raw sequenced data (fastq format), quality control and sequence statistics were performed by FastQC version 0.10.1 (http://www.bioinformatics.babraham.ac.uk/projects/fastqc/). After trimming the low quality bases using in-house built script, the spliced mapping process was performed by TopHat for each sample against to both genome and transcriptome references. Finally, SAMtools (version 0.1.18) and Linux Shell were used to extract mapped reads and other statistical information. Gene expression levels were calculated based on read counts which also included unambiguous mapped reads according to negative binomial distribution. FPKM (Fragments per kilobase of exon per million fragments mapped) values represent expression level of both gene and transcript.

### Validation of sequencing data by real-time quantitative PCR

Validation of sequencing data for five randomly selected miRNAs was performed by real-time quantitative PCR (Q-PCR). The stem-loop primer and Q-PCR pairs are summarized in Additional file 5. The cDNA was synthesized from 1 μg of total RNA using a PrimeScript RT reagent Kit (Takara, Dalian, China). Q-PCR was performed using a ABI7500 (Applied Biosystems, CA, USA) and a standardized protocol. In a 20 μl reaction mixture, 2.0 μl of cDNA (at a 1:4 dilution) was used for amplification, with 10 μl of SYBR Premix Ex TaqTM II (TaKaRa), 1.6 μl of a miR-specific Primer set (0.8 μl of each forward and reverse primer), 0.4 μl ROX Dye II and 6 μl of water. The reactions were incubated at 95°C for 30 s, followed by 40 cycles of 95°C for 5s, 60°C for 34 s. The abundance of selected miRNAs was normalized relative to that of U6 snRNA. All reactions were performed in triplicate. The threshold cycle (CT) was determined using the default threshold settings and the data was analyzed using the 2^–ΔΔCt^ method [60].

### miRNA target prediction, analysis of expression correlation and enriched KEGG pathway annotation

The 3′-UTR sequences were extracted according to gene annotation information. Potential targets for miRNAs were predicted using the miRanda, RNAhybrid and TargetScan algorithms simultaneously. Parameters were set as follows: score threshold at 50, energy threshold at -20 kcal/mol, scaling parameter at 4 and gap-open penalty at -2 for miRanda; helix constraint at 2–8, max bulge loop size at 3, max internal loop size at 3 and energy threshold at -20 kcal/mol for RNAhybrid; default parameters were used for TargetScan. The overlapping target genes were selected for further analysis. Correlation between the differentially expressed miRNAs and expression of their target genes was analyzed using R software. The KEGG pathways were used to perform pathway annotation of miRNA targets. The enrichment *p* values were calculated using hypergeometric distribution.

### piRNA predication

After removing miRNA tags, tag lengths ranging between 24 to 33 nt were simultaneously aligned to sheep genomic sequences and known piRNAs sequences in piRNABank [44] using bowtie [61] allowing two mismatches. The mapped reads (genome hits <20) were used to analyze piRNAs. Simultaneously, piRNA sequences and piRNA clusters were also predicted by a *de novo* method using proTRAC software [62].

## Electronic supplementary material

Additional file 1: **Distribution of total small RNA reads and tags by Solexa sequencing.** (DOC 100 KB)

Additional file 2: **Associations of expressions between the differentially expressed miRNAs and their target genes.** (DOC 610 KB)

Additional file 3: **Gonadal hormone secretion related pathways in which target genes of differentially expressed miRNAs enriched in this study.** The pathway maps were downloaded in KEGG database (http://www.genome.jp/kegg/). (TIFF 81 KB)

Additional file 4: **Gonadotropin secretion related pathways in which target genes of differentially expressed miRNAs enriched and their relationship.** The pathway map was downloaded in KEGG database (http://www.genome.jp/kegg/). (TIFF 56 KB)

Additional file 5: **Primer sequences for reverse transcription and Q-PCR of five randomly selected miRNAs.** (DOC 41 KB)

## Abbreviations

sRNAs: Small RNAs
STH: Small tail Han (sheep)
FSH: Follicle-stimulating hormone
LH: Luteinizing hormone
TSA: Tan ewes at anestrus in spring
TAL: Tan ewes at luteal phase in autumn
TAP: Tan ewes at proestrus in autumn
TAE: Tan ewes at estrus in autumn
HSL: STH ewes at luteal phase in spring
HSP: STH ewes at proestrus in spring
HSE: STH ewes at estrus in spring
HAE: STH ewes at estrus in autumn.
