# Supplementary material for: Characterization and comparative profiling of ovarian microRNAs during ovine anestrus and the breeding season
Source: BMC Genomics. 2014 Oct 15;15(1):899. doi: 10.1186/1471-2164-15-899 (PMC4287553; doi:10.1186/1471-2164-15-899)
Supplement: Supplementary file 1 — Additional file 1: Distribution of total small RNA reads and tags by Solexa sequencing. (DOC 100 KB) [file 12864_2014_6785_MOESM1_ESM.doc]

**The distribution of total small RNA reads and tags by Solexa sequencing**

|  | **TSA** | **TAL** | **TAP** | **TAE** | **HSL** | **HSP** | **HSE** | **HAE** |
| --- | --- | --- | --- | --- | --- | --- | --- | --- |
| Sequenced reads number | 13398411 | 13432260 | 43816873 | 32986664 | 13740629 | 11301741 | 10050475 | 17967117 |
| 3'adapter contaminants reads | 1245291 | 165189 | 2265888 | 91440 | 2178861 | 1153977 | 78398 | 25690 |
|  | 9.3% | 1.2% | 5.2% | 0.3% | 15.9% | 10.2% | 0.8% | 0.1% |
| Reads small than 17-nt or more than 35-nt | 3090397 | 2252564 | 2289379 | 8574621 | 2537733 | 1512681 | 406268 | 436037 |
|  | 23.1% | 16.8% | 5.2% | 26.0% | 18.5% | 13.4% | 4.0% | 2.4% |
| Clean reads number^N0^ | 9062723 | 11014507 | 39261606 | 24320603 | 9024035 | 8635083 | 9565809 | 17505390 |
|  | 67.64% | 82.00% | 89.60% | 73.73% | 65.67% | 76.40% | 95.18% | 97.43% |
| Mapped genome reads number^N1^ | 7049153 | 9254826 | 29610799 | 15951897 | 6096027 | 7600355 | 8487146 | 14839460 |
| N1/N0% | 77.78% | 84.02% | 75.42% | 65.59% | 67.55% | 88.02% | 88.72% | 84.77% |
| Mapped reads annotation |  |  |  |  |  |  |  |  |
| mRNA | 738114 | 164576 | 188567 | 1015795 | 138623 | 183986 | 31711 | 76000 |
| tRNA | 141370 | 2243128 | 12875064 | 3073691 | 747622 | 871326 | 1069461 | 8805364 |
| rRNA | 616351 | 338253 | 315802 | 3445119 | 188574 | 213976 | 59181 | 172045 |
| snoRNA | 1043491 | 119025 | 228028 | 1280117 | 956576 | 197939 | 160143 | 75390 |
| other sRNA in Rfam | 86764 | 41853 | 67401 | 81539 | 55232 | 40570 | 24436 | 24416 |
| Sum | 2626090 | 2906835 | 13674862 | 8896261 | 2086627 | 1507797 | 1344932 | 9153215 |
|  | 37.25% | 31.41% | 46.18% | 55.77% | 34.23% | 19.84% | 15.85% | 61.68% |
| Total microRNAs reads | 2759600 | 5441985 | 14623355 | 2618347 | 3558468 | 5374227 | 6400291 | 5073141 |
|  | 39.15% | 58.80% | 49.39% | 16.41% | 58.37% | 70.71% | 75.41% | 34.19% |
| Unknown reads | 1663463 | 906006 | 1312582 | 4437289 | 450932 | 718331 | 741923 | 613104 |
|  |  |  |  |  |  |  |  |  |
| Distinct reads number (tags) | 2026065 | 1094027 | 1981029 | 3545642 | 691984 | 698548 | 426405 | 376202 |
| Clean distinct reads number (tags)^N2^ | 1286873 | 569481 | 1232552 | 2302362 | 467282 | 421189 | 284609 | 254983 |
|  | 63.52% | 52.05% | 62.22% | 64.93% | 67.53% | 60.29% | 66.75% | 67.78% |
| Mapped genome tags number^N3^ | 1096586 | 277064 | 354877 | 1780306 | 199091 | 283200 | 115293 | 157567 |
| N3/N2% | 85.21% | 48.65% | 28.79% | 77.33% | 42.61% | 67.24% | 40.51% | 61.80% |
| Mapped tags annotation |  |  |  |  |  |  |  |  |
| mRNA | 246695 | 76876 | 86283 | 322690 | 49896 | 74536 | 15574 | 36752 |
| tRNA | 4024 | 10677 | 19986 | 9893 | 8746 | 7852 | 5974 | 6917 |
| rRNA | 15701 | 27185 | 32313 | 29379 | 15961 | 23493 | 13207 | 18509 |
| snoRNA | 35315 | 11967 | 16171 | 29184 | 13971 | 16070 | 9152 | 7881 |
| other sRNA in Rfam | 7372 | 5174 | 6418 | 4945 | 3955 | 5429 | 2537 | 2878 |
| miRNA tags | 12773 | 27512 | 37853 | 11104 | 30953 | 28518 | 27652 | 12689 |
| Unknown | 774706 | 117673 | 155853 | 1373111 | 75609 | 127302 | 41197 | 71941 |
| Total microRNAs | 202 | 349 | 370 | 237 | 302 | 350 | 323 | 366 |
| Known | 63 | 94 | 93 | 75 | 85 | 95 | 95 | 88 |
| Conserved | 136 | 246 | 267 | 159 | 205 | 244 | 233 | 182 |
| Novel | 3 | 9 | 10 | 3 | 12 | 10 | 10 | 8 |
