# Supplementary material for: Characterization and comparative profiling of ovarian microRNAs during ovine anestrus and the breeding season
Source: BMC Genomics. 2014 Oct 15;15(1):899. doi: 10.1186/1471-2164-15-899 (PMC4287553; doi:10.1186/1471-2164-15-899)
Supplement: Supplementary file 2 — Additional file 2: Associations of expressions between the differentially expressed miRNAs and their target genes. (DOC 610 KB) [file 12864_2014_6785_MOESM2_ESM.doc]

**Associations of expressions between the differentially expressed miRNAs and their target genes**

| **miRNA name** | **Gene ID** | **Gene name** | **Correlation coefficient** |
| --- | --- | --- | --- |
| miR-n-791 | CCG000507 | KAT2A | -0.992142134 |
| miR-n-77 | CCG017919 | WNT8B | -0.991999437 |
| miR-n-77 | CCG000683 | CACNG5 | -0.977291752 |
| miR-n-791 | CCG000146 | PIK3R5 | -0.973851681 |
| miR-n-789 | CCG010704 | NCSTN | -0.967550394 |
| miR-n-146 | CCG006828 | TAOK2 | -0.964303132 |
| miR-n-444 | CCG010704 | NCSTN | -0.953089499 |
| oar-miR-200a | CCG004410 | YLPM1 | -0.950149142 |
| miR-n-146 | CCG002755 | RASGRP2 | -0.944060493 |
| miR-n-77 | CCG001505 | MOS | -0.92120592 |
| miR-n-789 | CCG016021 | PYGB | -0.903218151 |
| miR-n-791 | CCG016021 | PYGB | -0.899237989 |
| miR-n-146 | CCG022439 | PRKACA | -0.896380177 |
| miR-n-146 | CCG008808 | NTF3 | -0.892733211 |
| miR-n-77 | CCG010167 | GNRHR2 | -0.880458984 |
| miR-n-77 | CCG012199 | GNG12 | -0.879979863 |
| miR-n-791 | CCG022615 | NOTCH3 | -0.875347051 |
| miR-n-77 | CCG010419 | ADCY5 | -0.875093401 |
| miR-n-77 | CCG006551 | BAIAP3 | -0.871014806 |
| miR-n-77 | CCG006615 | FZD9 | -0.869820018 |
| miR-n-146 | CCG020509 | RPS6KA1 | -0.868153684 |
| miR-n-77 | CCG015884 | STMN3 | -0.867689973 |
| miR-n-77 | CCG020683 | EPB49 | -0.862803507 |
| miR-n-444 | CCG022615 | NOTCH3 | -0.861477045 |
| miR-n-791 | CCG019037 | PSEN2 | -0.860454469 |
| miR-n-787 | CCG019037 | PSEN2 | -0.860454469 |
| miR-n-783 | CCG008763 | MAPK8IP2 | -0.857504506 |
| miR-n-789 | CCG008639 | EFCAB6 | -0.853922946 |
| miR-n-77 | CCG020554 | PLA2G2A | -0.853495251 |
| miR-n-791 | CCG000318 | G6PC3 | -0.852411465 |
| miR-n-791 | CCG008639 | EFCAB6 | -0.84959079 |
| miR-n-444 | CCG018841 | MEGF6 | -0.849343555 |
| miR-n-791 | CCG018841 | MEGF6 | -0.837403614 |
| miR-n-787 | CCG018841 | MEGF6 | -0.837403614 |
| miR-n-789 | CCG001916 | CAMK2G | -0.808275703 |
| miR-n-791 | CCG017361 | CACNA2D2 | -0.805387266 |
| miR-n-789 | CCG009394 | TENC1 | -0.787751637 |
| miR-n-789 | CCG004252 | ADCY4 | -0.782814676 |
| miR-n-791 | CCG018565 | DVL3 | -0.7822272 |
| miR-n-787 | CCG018565 | DVL3 | -0.7822272 |
| miR-n-77 | CCG018366 | FGFR1 | -0.77881883 |
| miR-n-791 | CCG000244 | MAP2K3 | -0.775649986 |
| miR-n-77 | CCG006836 | CACNA1H | -0.769674598 |
| miR-n-146 | CCG000785 | PROCA1 | -0.76739859 |
| miR-n-791 | CCG018155 | IKBKB | -0.764057551 |
| miR-n-146 | CCG003255 | FGFR3 | -0.754619749 |
| miR-n-791 | CCG011857 | INPP5D | -0.749644045 |
| miR-n-791 | CCG000443 | PIK3R6 | -0.74890478 |
| miR-n-791 | CCG021960 | CIB1 | -0.748606188 |
| miR-n-77 | CCG002568 | RELA | -0.733469405 |
| miR-n-783 | CCG012574 | RELB | -0.727186757 |
| miR-n-791 | CCG011446 | NTRK1 | -0.7252422 |
| miR-n-146 | CCG017955 | NFKB2 | -0.722925264 |
| miR-n-789 | CCG005853 | CABP7 | -0.722864401 |
| miR-n-791 | CCG019781 | CREB3 | -0.719519617 |
| miR-n-146 | CCG004297 | PLA2G4B | -0.718461945 |
| miR-n-791 | CCG012692 | PPP5C | -0.717071744 |
| miR-n-791 | CCG005853 | CABP7 | -0.713731366 |
| miR-n-791 | CCG017167 | RAF1 | -0.704614055 |
| miR-n-791 | CCG023218 | MAP2K2 | -0.700763978 |
| miR-n-789 | CCG023218 | MAP2K2 | -0.699745089 |
| miR-n-791 | CCG012562 | CBLC | -0.699716352 |
| miR-n-787 | CCG021620 | IGHA1 | -0.699713877 |
| miR-n-791 | CCG021620 | IGHA1 | -0.699713877 |
| miR-n-146 | CCG012692 | PPP5C | -0.691758286 |
| miR-n-791 | CCG004297 | PLA2G4B | -0.690200592 |
| miR-n-791 | CCG020551 | PLA2G2C | -0.689911917 |
| miR-n-791 | CCG006555 | TSC2 | -0.689165677 |
| miR-n-789 | CCG001040 | FLOT2 | -0.687877223 |
| miR-n-146 | CCG008962 | CACNA1B | -0.684523748 |
| miR-n-146 | CCG008838 | PLA2G6 | -0.682384464 |
| miR-n-77 | CCG000769 | FGF11 | -0.681440921 |
| miR-n-791 | CCG001040 | FLOT2 | -0.680798487 |
| miR-n-77 | CCG022266 | RASAL3 | -0.67973293 |
| miR-n-77 | CCG018006 | PPRC1 | -0.6726327 |
| miR-n-77 | CCG018595 | RALGPS2 | -0.668742115 |
| miR-n-791 | CCG017955 | NFKB2 | -0.666849374 |
| miR-n-146 | CCG000184 | CACNG4 | -0.665695717 |
| miR-n-791 | CCG009668 | MAPK12 | -0.661266084 |
| miR-n-77 | CCG020606 | WNT6 | -0.650651135 |
| miR-n-787 | CCG017297 | ZMYND10 | -0.650394268 |
| miR-n-146 | CCG005892 | MAPKAPK5 | -0.649656834 |
| miR-n-791 | CCG016062 | RBPJL | -0.640854094 |
| miR-n-77 | CCG008586 | CACNA1I | -0.637043254 |
| miR-n-146 | CCG017297 | ZMYND10 | -0.636664101 |
| miR-n-783 | CCG019022 | MAPKAPK2 | -0.636493575 |
| miR-n-786 | CCG010436 | NSUN4 | -0.633711628 |
| miR-n-791 | CCG005416 | DUSP22 | -0.632249117 |
| miR-n-444 | CCG011779 | PEAR1 | -0.630748157 |
| miR-n-789 | CCG011779 | PEAR1 | -0.629770717 |
| miR-n-77 | CCG003219 | HSPA8 | -0.62747763 |
| miR-n-77 | CCG007260 | IRS4 | -0.626770664 |
| miR-n-77 | CCG003352 | RPS6 | -0.624836245 |
| miR-n-791 | CCG008838 | PLA2G6 | -0.622025228 |
| miR-n-791 | CCG011779 | PEAR1 | -0.621720114 |
| miR-n-791 | CCG018560 | PRKCZ | -0.621265385 |
| miR-n-444 | CCG003316 | CTBP1 | -0.620807684 |
| miR-n-791 | CCG019475 | PLA2G2F | -0.618749906 |
| miR-n-783 | CCG019475 | PLA2G2F | -0.618749906 |
| miR-n-791 | CCG000841 | C17ORF46 | -0.6172134 |
| miR-n-789 | CCG019475 | PLA2G2F | -0.607807872 |
| miR-n-146 | CCG020154 | PLA2G2A | -0.605821391 |
| miR-n-77 | CCG011918 | MKNK1 | -0.604462855 |
| miR-n-146 | CCG007098 | UXT | -0.601987572 |
| miR-n-146 | CCG008165 | TNIK | -0.594084577 |
| miR-n-146 | CCG013441 | MAP4K1 | -0.588275544 |
| miR-n-789 | CCG000563 | EXOC7 | -0.584869709 |
| miR-n-791 | CCG000805 | CACNA1G | -0.582821037 |
| miR-n-791 | CCG007098 | UXT | -0.579821483 |
| miR-n-146 | CCG006557 | CCDC64B | -0.579787934 |
| miR-n-791 | CCG019933 | SPATA21 | -0.577350269 |
| miR-n-791 | CCG020154 | PLA2G2A | -0.574801249 |
| miR-n-789 | CCG006098 | PLA2G3 | -0.57427524 |
| miR-n-77 | CCG004489 | PLA2G4D | -0.573468467 |
| miR-n-791 | CCG012028 | MFNG | -0.572637127 |
| miR-n-444 | CCG011438 | DVL3 | -0.572453528 |
| miR-n-790 | CCG010398 | IFNAR2 | -0.570673594 |
| miR-n-791 | CCG012608 | CALM1 | -0.568725642 |
| miR-n-791 | CCG016707 | CAMK2B | -0.568037557 |
| miR-n-783 | CCG016707 | CAMK2B | -0.568037557 |
| miR-n-789 | CCG012028 | MFNG | -0.56498431 |
| miR-n-146 | CCG020560 | FDFT1 | -0.564918061 |
| miR-n-790 | CCG007082 | IL3RA | -0.562479854 |
| miR-n-791 | CCG000621 | WNT9B | -0.562182695 |
| miR-n-791 | CCG001067 | PLD2 | -0.556001258 |
| miR-n-791 | CCG009330 | CACNA1C | -0.553255199 |
| miR-n-791 | CCG018747 | PRDM16 | -0.54886043 |
| miR-n-791 | CCG000467 | GRAP | -0.546036462 |
| miR-n-787 | CCG002673 | TM7SF2 | -0.543499548 |
| miR-n-791 | CCG013073 | LHB | -0.54198993 |
| miR-n-791 | CCG000644 | RFNG | -0.540647664 |
| miR-n-791 | CCG006512 | PDGFA | -0.535776145 |
| miR-n-791 | CCG020777 | SH3BGRL3 | -0.532016531 |
| miR-n-791 | CCG002741 | RPS6KB2 | -0.531636494 |
| miR-n-789 | CCG022957 | PPARGC1B | -0.531244282 |
| miR-n-791 | CCG002490 | PPP1CA | -0.530741057 |
| miR-n-791 | CCG006557 | CCDC64B | -0.527167982 |
| miR-n-444 | CCG022396 | AES | -0.525647789 |
| miR-n-791 | CCG006432 | SH2B2 | -0.523936832 |
| miR-n-783 | CCG003034 | EGF | -0.522232968 |
| miR-n-791 | CCG008852 | TRAF2 | -0.522232968 |
| miR-n-791 | CCG022957 | PPARGC1B | -0.519373624 |
| miR-n-146 | CCG009464 | RAC2 | -0.51891184 |
| miR-n-146 | CCG006282 | PRKCB | -0.516227868 |
| miR-n-791 | CCG006534 | PDPK1 | -0.515490808 |
| miR-n-791 | CCG014918 | ARRB1 | -0.512406157 |
| miR-n-791 | CCG016817 | RHEB | -0.510019705 |
| miR-n-77 | CCG017273 | FLNB | -0.508777511 |
| miR-n-146 | CCG006512 | PDGFA | -0.506196376 |
| miR-n-77 | CCG009088 | WNT7B | -0.504019862 |
| miR-n-77 | CCG021958 | RASGRF1 | -0.504019862 |
| miR-n-77 | CCG008648 | CACNA2D4 | -0.504019862 |
| miR-n-77 | CCG002558 | CABP4 | -0.504019862 |
| miR-n-77 | CCG022309 | RAS | -0.504019862 |
| oar-miR-200a | CCG016381 | GNAI1 | -0.501739741 |
| miR-n-791 | CCG002554 | MAP3K11 | -0.499988087 |
| miR-n-791 | CCG009818 | MAP3K12 | -0.495026815 |
| miR-n-791 | CCG008713 | CRB2 | -0.493741931 |
| miR-n-77 | CCG009505 | CACNA2D4 | -0.484491168 |
| miR-n-791 | CCG015467 | MAP3K8 | -0.484410722 |
| miR-n-146 | CCG002673 | TM7SF2 | -0.483957939 |
| miR-n-791 | CCG002718 | BAD | -0.473259439 |
| miR-n-77 | CCG012633 | PRKCG | -0.466453026 |
| miR-n-77 | CCG019424 | CCDC21 | -0.465887594 |
| miR-n-791 | CCG002742 | FGF19 | -0.463419069 |
| miR-n-146 | CCG004485 | CHP | -0.455731268 |
| miR-n-146 | CCG002742 | FGF19 | -0.454537606 |
| miR-n-791 | CCG013473 | ADCY7 | -0.452910814 |
| miR-n-77 | CCG020311 | GLIPR2 | -0.452437103 |
| miR-n-789 | CCG022589 | TCF7 | -0.449103579 |
| miR-n-146 | CCG008863 | NPHP1 | -0.448542503 |
| miR-n-146 | CCG007049 | EBP | -0.445809872 |
| miR-n-783 | CCG010945 | MRAS | -0.445491812 |
| miR-n-789 | CCG013473 | ADCY7 | -0.441574221 |
| miR-n-77 | CCG019574 | TMEM39B | -0.441408803 |
| miR-n-791 | CCG008461 | RALGPS1 | -0.440043561 |
| miR-n-789 | CCG012835 | MC1R | -0.437656671 |
| miR-n-791 | CCG005742 | PRKAB1 | -0.43668154 |
| miR-n-783 | CCG009146 | FGF23 | -0.430119445 |
| miR-n-146 | CCG009505 | CACNA2D4 | -0.429736634 |
| miR-n-791 | CCG012835 | MC1R | -0.425374871 |
| miR-n-789 | CCG008380 | MFNG | -0.418225473 |
| miR-n-791 | CCG012823 | NTF4 | -0.417703498 |
| miR-n-444 | CCG012807 | NUMBL | -0.414676514 |
| miR-n-444 | CCG021303 | DLL1 | -0.414156763 |
| miR-n-146 | CCG009395 | SIX3 | -0.408002395 |
| miR-n-146 | CCG021958 | RASGRF1 | -0.408002395 |
| miR-n-146 | CCG022309 | RAS | -0.408002395 |
| miR-n-791 | CCG008380 | MFNG | -0.405514921 |
| miR-n-791 | CCG009147 | IL1R1 | -0.405346123 |
| miR-n-789 | CCG019505 | SORBS3 | -0.400740525 |
| miR-n-791 | CCG021303 | DLL1 | -0.400076276 |
| miR-n-146 | CCG000470 | SYNGR2 | -0.398824279 |
| miR-n-444 | CCG013282 | PSENEN | -0.392985664 |
| miR-n-146 | CCG000711 | CACNB1 | -0.387826915 |
| miR-n-786 | CCG003209 | UGT2C1 | -0.385709315 |
| miR-n-791 | CCG022786 | CAMK2A | -0.385253829 |
| miR-n-77 | CCG018028 | FGF8 | -0.384951788 |
| miR-n-77 | CCG009569 | FGF6 | -0.380395558 |
| miR-n-791 | CCG005042 | ITPR3 | -0.379870394 |
| miR-n-791 | CCG014915 | TTC36 | -0.366420884 |
| miR-n-444 | CCG010339 | APH1A | -0.36472004 |
| miR-n-77 | CCG006589 | ADCY9 | -0.362396105 |
| miR-n-791 | CCG014859 | CREB3L1 | -0.35951885 |
| miR-n-146 | CCG019589 | RAP1GAP | -0.356403724 |
| miR-n-791 | CCG009505 | CACNA2D4 | -0.356286774 |
| miR-n-77 | CCG008971 | ITPR2 | -0.354842783 |
| miR-n-146 | CCG009147 | IL1R1 | -0.351839849 |
| miR-n-791 | CCG000439 | SOCS3 | -0.351620253 |
| miR-n-791 | CCG017512 | GNAI2 | -0.35002871 |
| miR-n-790 | CCG003007 | KDR | -0.344903442 |
| miR-n-146 | CCG013006 | AKT2 | -0.342338784 |
| miR-n-791 | CCG013006 | AKT2 | -0.341557788 |
| miR-n-789 | CCG006714 | RPS6 | -0.340240626 |
| miR-n-791 | CCG022655 | HK3 | -0.336012486 |
| miR-n-146 | CCG003642 | FGF18 | -0.334733358 |
| miR-n-146 | CCG008239 | MAPK11 | -0.333625912 |
| miR-n-791 | CCG021958 | RASGRF1 | -0.333333333 |
| miR-n-791 | CCG016342 | ADCY1 | -0.333333333 |
| miR-n-787 | CCG009301 | CEL | -0.333333333 |
| miR-n-791 | CCG023166 | WNT3A | -0.333333333 |
| miR-n-791 | CCG003708 | RPS6 | -0.333333333 |
| miR-n-791 | CCG009088 | WNT7B | -0.333333333 |
| miR-n-444 | CCG006209 | DTX2 | -0.33188959 |
| miR-n-77 | CCG010562 | TEKT2 | -0.325168051 |
| miR-n-789 | CCG016342 | ADCY1 | -0.319410267 |
| miR-n-789 | CCG022309 | RAS | -0.319410267 |
| miR-n-791 | CCG009764 | PDGFB | -0.318808883 |
| miR-n-146 | CCG009886 | PRDM12 | -0.31606769 |
| miR-n-791 | CCG003638 | ADCY2 | -0.311723596 |
| miR-n-789 | CCG005751 | PPP1CC | -0.310230778 |
| miR-n-790 | CCG014127 | FLT3 | -0.309686991 |
| miR-n-783 | CCG017817 | DUSP5 | -0.309534469 |
| miR-n-77 | CCG022909 | CREB3L3 | -0.302904817 |
| miR-n-791 | CCG017613 | CACNA1D | -0.297848805 |
| miR-n-146 | CCG017495 | MAPKAPK3 | -0.297435256 |
| miR-n-783 | CCG005656 | CABP1 | -0.297297297 |
| miR-n-146 | CCG014915 | TTC36 | -0.294757392 |
| miR-n-146 | CCG010497 | DHCR24 | -0.29305923 |
| miR-n-444 | CCG013422 | DLL3 | -0.282749507 |
| miR-n-77 | CCG011751 | PLD1 | -0.280557306 |
| miR-n-77 | CCG000634 | SLC2A4 | -0.280507373 |
| miR-n-77 | CCG000711 | CACNB1 | -0.279203576 |
| miR-n-146 | CCG020548 | MAP3K2 | -0.277681584 |
| miR-n-791 | CCG003252 | LEF1 | -0.249712194 |
| miR-n-791 | CCG020672 | STMN4 | -0.245297221 |
| miR-n-791 | CCG006298 | FAM109A | -0.242453552 |
| miR-n-77 | CCG006557 | CCDC64B | -0.238892959 |
| miR-n-791 | CCG000814 | FASN | -0.237224122 |
| miR-n-791 | CCG004605 | TLE3 | -0.229343994 |
| miR-n-791 | CCG008470 | DUSP16 | -0.225635913 |
| miR-n-146 | CCG018928 | RASAL2 | -0.220615597 |
| miR-n-77 | CCG017507 | PRKAR2A | -0.219061626 |
| miR-n-791 | CCG012808 | GNA0 | -0.211070467 |
| miR-n-791 | CCG015701 | PYGB | -0.209311951 |
| miR-n-791 | CCG010562 | TEKT2 | -0.208461456 |
| miR-n-444 | CCG012697 | TLE2 | -0.193383104 |
| miR-n-791 | CCG006263 | PHKG1 | -0.189053307 |
| miR-n-787 | CCG012697 | TLE2 | -0.188287191 |
| miR-n-146 | CCG007772 | IKBKG | -0.18009718 |
| miR-n-77 | CCG019475 | PLA2G2F | -0.179446947 |
| miR-n-791 | CCG004747 | PCK2 | -0.178590939 |
| miR-n-77 | CCG005416 | DUSP22 | -0.178576777 |
| miR-n-783 | CCG012489 | SYNGR4 | -0.177168253 |
| miR-n-77 | CCG018747 | PRDM16 | -0.17650473 |
| miR-n-444 | CCG009417 | DTX3 | -0.174837342 |
| miR-n-789 | CCG011918 | MKNK1 | -0.174637289 |
| miR-n-146 | CCG016210 | EBP | -0.169395132 |
| miR-n-77 | CCG002549 | MAP4K2 | -0.163636114 |
| miR-n-77 | CCG006432 | SH2B2 | -0.160349302 |
| miR-n-77 | CCG008838 | PLA2G6 | -0.160044219 |
| miR-n-791 | CCG022504 | PDGFRB | -0.15178385 |
| miR-n-77 | CCG000235 | MPP3 | -0.150791627 |
| miR-n-77 | CCG011529 | PRKAB2 | -0.144561383 |
| miR-n-77 | CCG002392 | NAV2 | -0.132954931 |
| miR-n-444 | CCG021641 | DLK1 | -0.126011137 |
| miR-n-146 | CCG022931 | FGFR4 | -0.117723566 |
| miR-n-791 | CCG002240 | DUSP8 | -0.10579239 |
| miR-n-146 | CCG007127 | FLNA | -0.102778275 |
| miR-n-77 | CCG006555 | TSC2 | -0.091617792 |
| miR-n-77 | CCG008909 | DAB2IP | -0.080488651 |
| miR-n-77 | CCG001059 | SREBF1 | -0.080320805 |
| miR-n-77 | CCG005026 | RPS6 | -0.077795363 |
| miR-n-789 | CCG011006 | PTPRF | -0.042771176 |
| miR-n-791 | CCG022984 | SHC2 | -0.042556313 |
| oar-miR-200a | CCG005694 | ANAPC7 | -0.035140384 |
| miR-n-791 | CCG011006 | PTPRF | -0.035121413 |
| miR-n-789 | CCG020751 | TLE1 | -0.034508787 |
| miR-n-791 | CCG007127 | FLNA | -0.03031187 |
| miR-n-77 | CCG002737 | FZD4 | -0.026085808 |
| miR-n-791 | CCG022399 | WNT9A | -0.023708934 |
| miR-n-787 | CCG020276 | CYP27C1 | -0.022490373 |
| miR-n-77 | CCG000767 | NF1 | -0.018206293 |
| miR-n-791 | CCG011585 | TCTEX1D4 | -0.015911351 |
| miR-n-789 | CCG001071 | PER1 | -0.008968746 |
| miR-n-791 | CCG005692 | DTX1 | 0.010307473 |
| miR-n-77 | CCG019933 | SPATA21 | 0.020529815 |
| miR-n-146 | CCG022620 | CACNA1A | 0.043596783 |
| miR-n-791 | CCG017377 | DUSP7 | 0.052715418 |
| miR-n-791 | CCG005293 | DAXX | 0.053396687 |
| miR-n-146 | CCG007209 | ELK1 | 0.073570066 |
| miR-n-789 | CCG004190 | DLL4 | 0.074428756 |
| miR-n-444 | CCG004190 | DLL4 | 0.079506179 |
| miR-n-77 | CCG022451 | MUC16 | 0.079744036 |
| miR-n-791 | CCG018366 | FGFR1 | 0.085045587 |
| miR-n-783 | CCG018362 | DUSP4 | 0.086212267 |
| miR-n-77 | CCG018676 | CACNA1S | 0.090013533 |
| miR-n-146 | CCG020554 | PLA2G2A | 0.093968262 |
| miR-n-789 | CCG008350 | CSNK1E | 0.106364445 |
| miR-n-791 | CCG005915 | NCOR2 | 0.109166442 |
| miR-n-791 | CCG005923 | TESC | 0.110804149 |
| miR-n-791 | CCG009070 | GYS2 | 0.116510346 |
| miR-n-77 | CCG018560 | PRKCZ | 0.122145644 |
| miR-n-77 | CCG011857 | INPP5D | 0.125395547 |
| miR-n-146 | CCG006551 | BAIAP3 | 0.127760191 |
| miR-n-791 | CCG011107 | JUN | 0.142133235 |
| miR-n-77 | CCG020154 | PLA2G2A | 0.165351053 |
| miR-n-791 | CCG020554 | PLA2G2A | 0.169552119 |
| miR-n-146 | CCG011107 | JUN | 0.170984625 |
| miR-n-146 | CCG005978 | KSR2 | 0.181145813 |
| miR-n-77 | CCG001882 | MAPK8 | 0.19052758 |
| miR-n-791 | CCG020606 | WNT6 | 0.193115754 |
| miR-n-146 | CCG010382 | RAP1A | 0.196320915 |
| miR-n-77 | CCG000916 | MAP3K3 | 0.196869022 |
| miR-n-791 | CCG015539 | CACNB2 | 0.206746374 |
| miR-n-77 | CCG005663 | MAPK1 | 0.224112976 |
| miR-n-77 | CCG019781 | CREB3 | 0.24669167 |
| miR-n-77 | CCG004297 | PLA2G4B | 0.248731064 |
| miR-n-77 | CCG007098 | UXT | 0.257575431 |
| miR-n-791 | CCG020236 | WNT10A | 0.269578695 |
| miR-n-789 | CCG006442 | LFNG | 0.281632325 |
| miR-n-791 | CCG006442 | LFNG | 0.29195322 |
| miR-n-146 | CCG006804 | SDR42E2 | 0.293019143 |
| miR-n-789 | CCG001921 | EIF4EBP2 | 0.296270798 |
| miR-n-77 | CCG004496 | EFCAB11 | 0.29939912 |
| miR-n-791 | CCG010911 | FGF12 | 0.302174808 |
| miR-n-77 | CCG015643 | PLCB4 | 0.314198877 |
| miR-n-146 | CCG018861 | DUSP10 | 0.325351123 |
| miR-n-77 | CCG011585 | TCTEX1D4 | 0.330528432 |
| miR-n-77 | CCG013006 | AKT2 | 0.335633387 |
| miR-n-783 | CCG018676 | CACNA1S | 0.343852031 |
| miR-n-77 | CCG015701 | PYGB | 0.357643617 |
| miR-n-789 | CCG002381 | TMEM135 | 0.401585798 |
| miR-n-77 | CCG015443 | STK4 | 0.405479974 |
| miR-n-77 | CCG000563 | EXOC7 | 0.410493666 |
| miR-n-77 | CCG005246 | MAPK14 | 0.41332171 |
| miR-n-77 | CCG015688 | ASIP | 0.419785728 |
| miR-n-77 | CCG016707 | CAMK2B | 0.44081612 |
| miR-n-77 | CCG000378 | WNT3 | 0.445664377 |
| miR-n-77 | CCG016466 | TNS3 | 0.448234219 |
| miR-n-77 | CCG012730 | GYS1 | 0.448661393 |
| miR-n-444 | CCG022858 | TLE6 | 0.45060429 |
| miR-n-790 | CCG018608 | IL10 | 0.451443471 |
| miR-n-77 | CCG002741 | RPS6KB2 | 0.460613193 |
| miR-n-77 | CCG015641 | PPCK1 | 0.463131766 |
| miR-n-77 | CCG007209 | ELK1 | 0.463281695 |
| miR-n-791 | CCG014931 | HPX | 0.466432009 |
| miR-n-791 | CCG022654 | PPARGC1B | 0.467422387 |
| miR-n-789 | CCG022858 | TLE6 | 0.473617038 |
| miR-n-77 | CCG000469 | PRKCA | 0.47542389 |
| miR-n-791 | CCG000683 | CACNG5 | 0.475815645 |
| miR-n-77 | CCG017377 | DUSP7 | 0.480362703 |
| miR-n-77 | CCG002718 | BAD | 0.483300525 |
| miR-n-77 | CCG019022 | MAPKAPK2 | 0.488903055 |
| miR-n-77 | CCG002742 | FGF19 | 0.492012586 |
| miR-n-77 | CCG020509 | RPS6KA1 | 0.493156972 |
| miR-n-77 | CCG000421 | MAP2K6 | 0.498373895 |
| miR-n-787 | CCG014817 | DTX4 | 0.500555659 |
| miR-n-77 | CCG023000 | MKNK2 | 0.50062301 |
| miR-n-146 | CCG021605 | CIB2 | 0.522990678 |
| miR-n-77 | CCG008163 | CACNG2 | 0.52772565 |
| miR-n-791 | CCG016597 | PEG10 | 0.531114786 |
| miR-n-77 | CCG003255 | FGFR3 | 0.536465055 |
| miR-n-77 | CCG000318 | G6PC3 | 0.537427528 |
| miR-n-146 | CCG017780 | PDZD7 | 0.541671647 |
| miR-n-146 | CCG013372 | CACNG7 | 0.542378722 |
| miR-n-791 | CCG016930 | FZD2 | 0.545522961 |
| miR-n-791 | CCG010739 | SHC1 | 0.545771472 |
| miR-n-77 | CCG000439 | SOCS3 | 0.551666933 |
| miR-n-77 | CCG019756 | MAP3K6 | 0.559881249 |
| miR-n-783 | CCG000916 | MAP3K3 | 0.560648971 |
| miR-n-791 | CCG008909 | DAB2IP | 0.573120424 |
| miR-n-146 | CCG017369 | TRANK1 | 0.573458834 |
| miR-n-77 | CCG019605 | G6PC2 | 0.577706998 |
| miR-n-791 | CCG021605 | CIB2 | 0.578660437 |
| miR-n-77 | CCG012692 | PPP5C | 0.585354213 |
| miR-n-787 | CCG019895 | SIGMAR1 | 0.595191821 |
| miR-n-791 | CCG000767 | NF1 | 0.600357671 |
| miR-n-77 | CCG002490 | PPP1CA | 0.601776663 |
| miR-n-790 | CCG017521 | CX3CR1 | 0.613638461 |
| miR-n-77 | CCG012993 | PPM1N | 0.614923102 |
| miR-n-77 | CCG009146 | FGF23 | 0.618007123 |
| miR-n-77 | CCG009886 | PRDM12 | 0.627757101 |
| miR-n-77 | CCG018992 | PIK3CD | 0.627780647 |
| miR-n-77 | CCG001067 | PLD2 | 0.629316992 |
| miR-n-77 | CCG011438 | DVL3 | 0.642810334 |
| miR-n-790 | CCG002385 | CLCF1 | 0.644259055 |
| miR-n-77 | CCG016012 | ASIP | 0.64452427 |
| miR-n-77 | CCG010425 | CREB3L4 | 0.653388834 |
| miR-n-77 | CCG009147 | IL1R1 | 0.654328389 |
| miR-n-791 | CCG001075 | MAP3K14 | 0.675340913 |
| miR-n-77 | CCG022940 | CD14 | 0.67741948 |
| miR-n-77 | CCG005923 | TESC | 0.677777809 |
| miR-n-77 | CCG002650 | CABP2 | 0.694654267 |
| miR-n-77 | CCG002453 | RPS6KA4 | 0.707821094 |
| miR-n-791 | CCG008249 | SOS1 | 0.708403054 |
| miR-n-783 | CCG008249 | SOS1 | 0.708403054 |
| miR-n-791 | CCG017919 | WNT8B | 0.727606875 |
| miR-n-791 | CCG000875 | B3GNTL1 | 0.733779292 |
| miR-n-77 | CCG006263 | PHKG1 | 0.743650278 |
| miR-n-791 | CCG006025 | CRKL | 0.747216393 |
| miR-n-77 | CCG017959 | MYOF | 0.756965664 |
| miR-n-146 | CCG002392 | NAV2 | 0.759168547 |
| miR-n-791 | CCG006677 | SYNGR3 | 0.759470552 |
| miR-n-77 | CCG005109 | MAPK13 | 0.766507722 |
| miR-n-791 | CCG008398 | ADCY6 | 0.766528004 |
| miR-n-791 | CCG014831 | ARHGEF17 | 0.767218561 |
| miR-n-787 | CCG010861 | SNED1 | 0.769154471 |
| miR-n-77 | CCG016021 | PYGB | 0.771348256 |
| miR-n-791 | CCG019540 | PRKAG3 | 0.772677216 |
| miR-n-77 | CCG007756 | ARAF | 0.773395568 |
| miR-n-77 | CCG000184 | CACNG4 | 0.774427438 |
| miR-n-146 | CCG006677 | SYNGR3 | 0.783308585 |
| miR-n-146 | CCG005783 | TAOK3 | 0.784770716 |
| miR-n-791 | CCG021083 | SASH1 | 0.787119115 |
| miR-n-146 | CCG022007 | GOLGA5 | 0.78963969 |
| miR-n-77 | CCG017910 | SORBS1 | 0.795523424 |
| miR-n-77 | CCG006298 | FAM109A | 0.810826839 |
| miR-n-146 | CCG018540 | PTPN7 | 0.8119438 |
| miR-n-791 | CCG016711 | CREB3L2 | 0.815955628 |
| miR-n-77 | CCG020022 | PLA2G2E | 0.817350454 |
| miR-n-791 | CCG017856 | PPP1R3C | 0.819038052 |
| miR-n-77 | CCG017361 | CACNA2D2 | 0.821320948 |
| miR-n-77 | CCG000621 | WNT9B | 0.825432435 |
| miR-n-77 | CCG006098 | PLA2G3 | 0.834477251 |
| miR-n-791 | CCG022597 | EIF4E1B | 0.84231173 |
| miR-n-789 | CCG015694 | SRC | 0.843435284 |
| miR-n-77 | CCG022439 | PRKACA | 0.844208399 |
| miR-n-783 | CCG015694 | SRC | 0.845968635 |
| miR-n-791 | CCG000235 | MPP3 | 0.846115014 |
| miR-n-77 | CCG012608 | CALM1 | 0.846634826 |
| miR-n-791 | CCG017507 | PRKAR2A | 0.861231293 |
| miR-n-791 | CCG015386 | PPP1R3D | 0.862894606 |
| miR-n-791 | CCG021231 | MAP3K4 | 0.866670139 |
| miR-n-783 | CCG021231 | MAP3K4 | 0.866670139 |
| miR-n-791 | CCG006015 | MAML3 | 0.867239965 |
| miR-n-789 | CCG014615 | CRY2 | 0.872835983 |
| miR-n-77 | CCG000805 | CACNA1G | 0.875667824 |
| miR-n-146 | CCG022387 | RASGRF2 | 0.880504482 |
| miR-n-77 | CCG017495 | MAPKAPK3 | 0.883478879 |
| miR-n-77 | CCG017430 | ITIH4 | 0.886510869 |
| miR-n-791 | CCG011280 | NGF | 0.888913661 |
| miR-n-77 | CCG012835 | MC1R | 0.892693147 |
| miR-n-77 | CCG005956 | FZD10 | 0.912504474 |
| miR-n-77 | CCG017512 | GNAI2 | 0.912853143 |
| miR-n-77 | CCG008852 | TRAF2 | 0.924009513 |
| miR-n-791 | CCG012199 | GNG12 | 0.92919276 |
| miR-n-791 | CCG019424 | CCDC21 | 0.932759319 |
| miR-n-77 | CCG002554 | MAP3K11 | 0.933010341 |
| miR-n-444 | CCG000651 | ZNF385C | 0.935351358 |
| miR-n-791 | CCG000769 | FGF11 | 0.939458574 |
| miR-n-146 | CCG006529 | HSPB1 | 0.942173357 |
| miR-n-787 | CCG000651 | ZNF385C | 0.946694215 |
| miR-n-146 | CCG004240 | PLA2G4F | 0.950274233 |
| miR-n-791 | CCG004240 | PLA2G4F | 0.950586376 |
| miR-n-77 | CCG008991 | CACNB3 | 0.956570162 |
| miR-n-77 | CCG018574 | PRDM16 | 0.956775103 |
| miR-n-146 | CCG018953 | CACNA1E | 0.958142631 |
| miR-n-146 | CCG007762 | FLNA | 0.9591418 |
| miR-n-77 | CCG008994 | NAV3 | 0.961900334 |
| miR-n-791 | CCG022614 | MAP2K7 | 0.961916096 |
| miR-n-77 | CCG012562 | CBLC | 0.962678202 |
| miR-n-791 | CCG006740 | CREBBP | 0.965090439 |
| miR-n-146 | CCG006836 | CACNA1H | 0.966643347 |
| miR-n-146 | CCG011245 | RASA2 | 0.967393058 |
| miR-n-791 | CCG018218 | PPP1R3B | 0.968900563 |
| miR-n-146 | CCG009081 | SOAT2 | 0.972744341 |
| miR-n-791 | CCG018953 | CACNA1E | 0.977107124 |
| miR-n-77 | CCG014211 | FGF9 | 0.977432527 |
| miR-n-77 | CCG015735 | TOMM34 | 0.977899485 |
| miR-n-791 | CCG006836 | CACNA1H | 0.983444197 |
| miR-n-146 | CCG008586 | CACNA1I | 0.98870046 |
| miR-n-791 | CCG008586 | CACNA1I | 0.989709801 |
| miR-n-146 | CCG005206 | TNF | 0.993689317 |
| miR-n-791 | CCG013392 | ZNF296 | 0.993999089 |
| miR-n-146 | CCG013392 | ZNF296 | 0.996742762 |
| miR-n-77 | CCG022709 | GADD45B | 0.997158089 |
| miR-n-146 | CCG004489 | PLA2G4D | 0.997368647 |
| miR-n-146 | CCG022626 | GNG12 | 0.997555946 |
| miR-n-791 | CCG022626 | GNG12 | 0.999744098 |
| miR-n-791 | CCG018235 | RPS6 | 1 |
| miR-n-77 | CCG019311 | PLA2G5 | / |
| miR-n-77 | CCG002315 | TYR | / |
| miR-n-146 | CCG018073 | LIPN | / |
| miR-n-789 | CCG002315 | TYR | / |
| miR-n-789 | CCG001036 | G6PC | / |
