# Supplementary figures and images for: Characterization and comparative profiling of ovarian microRNAs during ovine anestrus and the breeding season
Source: BMC Genomics. 2014 Oct 15;15(1):899. doi: 10.1186/1471-2164-15-899 (PMC4287553; doi:10.1186/1471-2164-15-899)

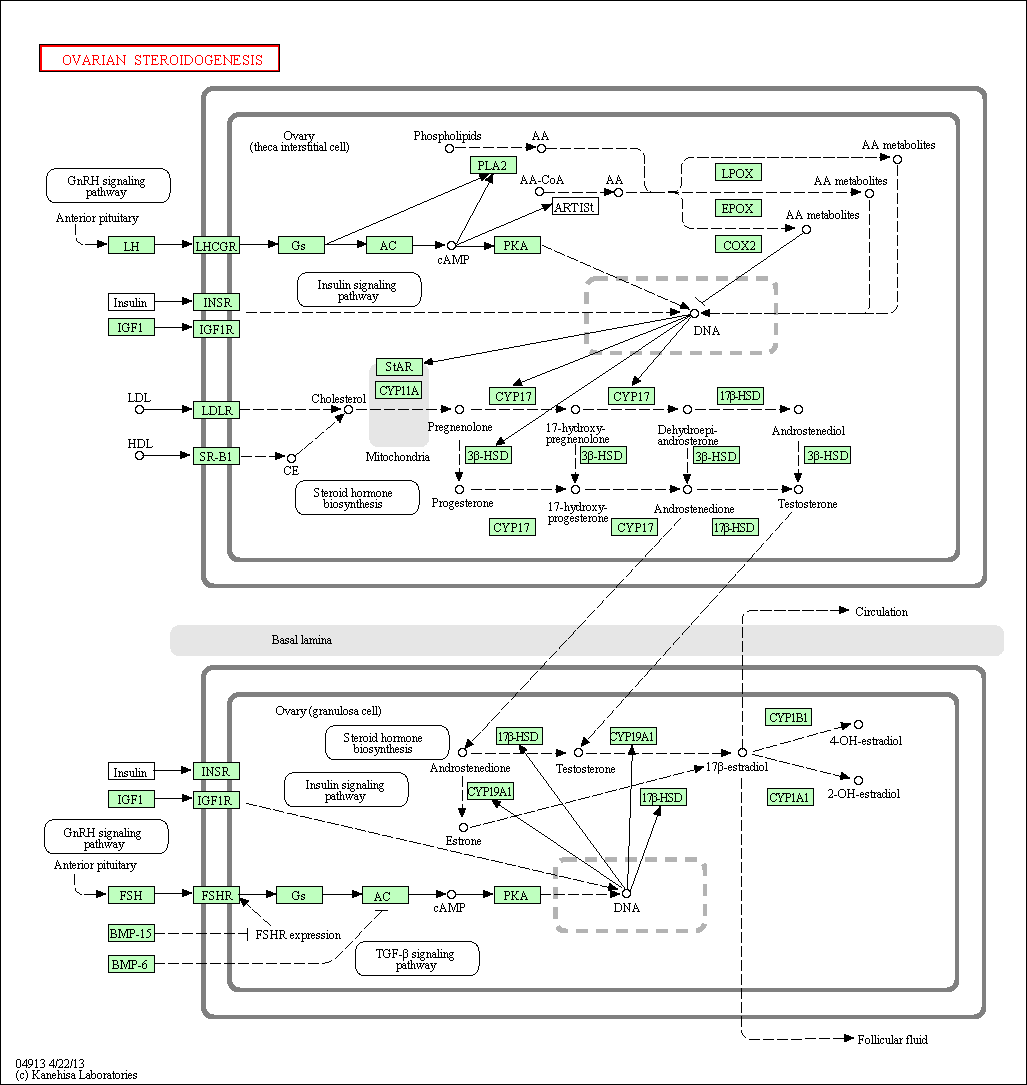

Supplement: Supplementary file 3 — Additional file 3: Gonadal hormone secretion related pathways in which target genes of differentially expressed miRNAs enriched in this study. The pathway maps were downloaded in KEGG database (http://www.genome.jp/kegg/). (TIFF 81 KB) [file 12864_2014_6785_MOESM3_ESM.tiff]

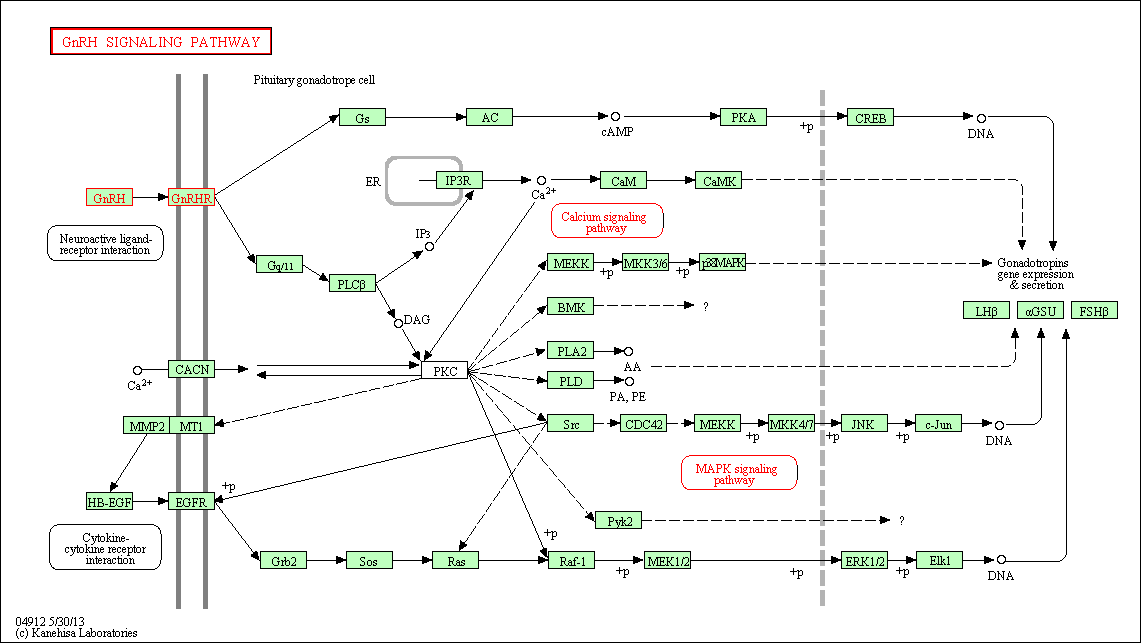

Supplement: Supplementary file 4 — Additional file 4: Gonadotropin secretion related pathways in which target genes of differentially expressed miRNAs enriched and their relationship. The pathway map was downloaded in KEGG database (http://www.genome.jp/kegg/). (TIFF 56 KB) [file 12864_2014_6785_MOESM4_ESM.tiff]
