# Supplementary material for: Characterization and comparative profiling of ovarian microRNAs during ovine anestrus and the breeding season
Source: BMC Genomics. 2014 Oct 15;15(1):899. doi: 10.1186/1471-2164-15-899 (PMC4287553; doi:10.1186/1471-2164-15-899)
Supplement: Supplementary file 5 — Additional file 5: Primer sequences for reverse transcription and Q-PCR of five randomly selected miRNAs. (DOC 41 KB) [file 12864_2014_6785_MOESM5_ESM.doc]

**Primer sequences for reverse transcription and Q-PCR of five randomly selected miRNAs**

| **Names of miRNAs** | **Classes of primer** | **Primer sequences** |
| --- | --- | --- |
| miR-n-13 |  |  |
|  | Stem-loop primer | GTCGTATCCAGTGCAGGGTCCGAGGTATTCGCACTGGATACGAC ACAACCAG |
|  | Forward primer | CGGCTGGCAGTGTCTTAG |
|  | Reverse primer | GCAGGGTCCGAGGTAT |
| miR-n-162 |  |  |
|  | Stem-loop primer | GTCGTATCCAGTGCAGGGTCCGAGGTATTCGCACTGGATACGAC CGCCCAAT |
|  | Forward primer | CGGGCATCAACAGACATTA |
|  | Reverse primer | GCAGGGTCCGAGGTATT |
| miR-n-180 |  |  |
|  | Stem-loop primer | GTCGTATCCAGTGCAGGGTCCGAGGTATTCGCACTGGATACGAC TCAGTCTC |
|  | Forward primer | CGGGGCGTTATAAAGC |
|  | Reverse primer | GCAGGGTCCGAGGTA |
| oar-miR-374b |  |  |
|  | Stem-loop primer | GTCGTATCCAGTGCAGGGTCCGAGGTATTCGCACTGGATACGAC ACTTAGCA |
|  | Forward primer | AGCGGGGCATATAATA |
|  | Reverse primer | GCAGGGTCCGAGGTA |
| miR-n-17 |  |  |
|  | Stem-loop primer | GTCGTATCCAGTGCAGGGTCCGAGGTATTCGCACTGGATACGAC TCATTCCA |
|  | Forward primer | CGGCTTATTGGCACG |
|  | Reverse primer | GCAGGGTCCGAGGTAT |
